# Single-Exosome Profiling Identifies ITGB3+ and ITGAM+ Exosome Subpopulations as Promising Early Diagnostic Biomarkers and Therapeutic Targets for Colorectal Cancer

**DOI:** 10.34133/research.0041

**Published:** 2023-01-30

**Authors:** Wei Guo, Yanling Cai, Xianming Liu, Yuge Ji, Cuiyu Zhang, Liyan Wang, Wenting Liao, Yuefei Liu, Nan Cui, Jinsheng Xiang, Zesong Li, Di Wu, Jingxin Li

**Affiliations:** ^1^ Department of Physiology, School of Basic Medical Sciences, Department of Colorectal Surgery, Qilu Hospital, Cheeloo College of Medicine, Shandong University, Jinan, Shandong, 250012, China.; ^2^ Guangdong Provincial Key Laboratory of Systems Biology and Synthetic Biology for Urogenital Tumors, Department of Urology, The First Affiliated Hospital of Shenzhen University, Shenzhen Second People's Hospital (Shenzhen Institute of Translational Medicine), Shenzhen, 518035, China.; ^3^ Shenzhen Key Laboratory of Genitourinary Tumor, Department of Urology, The First Affiliated Hospital of Shenzhen University, Shenzhen Second People's Hospital (Shenzhen Institute of Translational Medicine), Shenzhen, 518035, China.; ^4^ Department of Gastrointestinal Surgery, Shenzhen People’s Hospital, The Second Clinical Medical College of Jinan University, The First Affiliated Hospital of Southern University of Science and Technology, Shenzhen, Guangdong, 518020, China.; ^5^ Center for Experimental Nuclear Medicine and Electron Microscope, School of Basic Medical Sciences, Shandong University, Jinan, Shandong, 250012, China.; ^6^ Shenzhen SecreTech Co., Ltd., Shenzhen, China.; ^7^ Vesicode AB, Nobelsvag 16, Solna, Sweden.

## Abstract

Tumor metastasis is a hallmark of colorectal cancer (CRC), in which exosome plays a crucial role with its function in intercellular communication. Plasma exosomes were collected from healthy control (HC) donors, localized primary CRC and liver-metastatic CRC patients. We performed proximity barcoding assay (PBA) for single-exosome analysis, which enabled us to identify the alteration in exosome subpopulations associated with CRC progression. By in vitro and in vivo experiments, the biological impact of these subpopulations on cancer proliferation, migration, invasion, and metastasis was investigated. The potential application of exosomes as diagnostic biomarkers was evaluated in 2 independent validation cohorts by PBA. Twelve distinct exosome subpopulations were determined. We found 2 distinctly abundant subpopulations: one ITGB3-positive and the other ITGAM-positive. The ITGB3-positive cluster is rich in liver-metastatic CRC, compared to both HC group and primary CRC group. On the contrary, ITGAM-positive exosomes show a large-scale increase in plasma of HC group, compared to both primary CRC and metastatic CRC groups. Notably, both discovery cohort and validation cohort verified ITGB3+ exosomes as potential diagnostic biomarker. ITGB3+ exosomes promote proliferation, migration, and invasion capability of CRC. In contrast, ITGAM+ exosomes suppress CRC development. Moreover, we also provide evidence that one of the sources of ITGAM+ exosomes is macrophage. ITGB3+ exosomes and ITGAM+ exosomes are proven 2 potential diagnostic, prognostic, and therapeutic biomarkers for management of CRC.

## What is already known on this topic?

Previous studies have showed that exosomes play an important role in regulating proliferation, metastasis, chemoresistance, and recrudescence of CRC. The main characteristic of exosome is high degree of heterogeneity in molecular composition due to different origins. It is of great significance to study exosome individually because heterogeneity is missing in bulk-level analysis. In this study, we utilized proximity barcoding assay, the novel high-throughput method for single-exosome analysis to study CRC-related exosome subpopulations.

## What this study adds?

We found 2 differentially abundant exosome clusters by single-exosome analysis: ITGB3-positive and ITGAM-positive exosome subpopulation. In vitro and in vivo experiments proved that ITGB3+ exosomes promote proliferation, migration, and invasion of CRC. In contrast, ITGAM+ exosomes suppress CRC development. Moreover, we also provide evidence that one of the sources of ITGAM+ exosomes is macrophage.

## How this study might affect research, practice, or policy?

ITGB3+ exosomes and ITGAM+ exosomes are promising diagnostic and therapeutic target spots for early CRC management. Also, proximity barcoding assay will be a promising tool for liquid biopsy for all cancer types.

## Introduction

Colorectal cancer (CRC) is a common disease with a high incidence all over the world. When diagnosed with CRC, approximately 70.4% of patients have peripheral metastasis, mainly to lymph node, while 12.5% patients have distant metastasis of cancer, including liver metastasis. Five-year survival rate is 90% in localized primary CRC patients but decreased to 71% for peripheral metastatic CRC patients and 14% for patients with metastasis in distant organs [1]. CRC metastasis dramatically reduces postoperative survival rate; however, advanced CRC screening, diagnosis, and therapeutic methods are still under investigation [2].

Exosomes have attracted considerable attention from both research fields and biomedical industries because of the increasingly recognized physical and pathological functions and their potentials in diagnostic and therapeutic applications [3–5]. An exosome is a membrane-enveloped extracellular vesicle (EV) whose diameters ranging from 30 to 150 nm. Cells release exosomes to transmit biological intercellular information [6]. Exosomes are reported to play an important role in cancer metastasis and therefore are potential targets for liquid biopsy [7,8]. Exosomes are important in regulating CRC proliferation, metastasis, chemoresistance, and recrudescence in several studies. Exosomes originated from SW680 or SW640 promote angiogenesis [8]. Moreover, exosomes from CT26 dramatically promoted tumor proliferation and reduced CRC survival rate [9]. In addition, serum exosomal miR-203 could induce tumor-associated macrophages activation and promote liver metastasis of CRC [10]. Another previous study revealed that cancer-associated fibroblasts produce exosomes with high levels of oncogene miR-21, which led to increases AKT phosphorylation after transferring to CRC cells and was tightly correlated with chemoresistance to oxaliplatin [11].

Exosomes are highly heterogeneous in molecular composition owing to different stimuli from the microenvironment and cell origins. The membrane proteins of exosomes reflect the donor cell type, facilitating specific targeting of exosome vesicles to perform physiological or pathological roles on their recipient cells [12–16]. When it comes to exosome analysis of body fluids for the purposes of liquid biopsy or therapeutic applications, heterogeneity of exosomes is especially critical. It is meaningful to study exosome individually because heterogeneity is missing in bulk-level analyses like enzyme-linked immunosorbent assay or mass spectroscopy. Here, we utilized proximity barcoding assay (PBA) [17], an innovative and fast high-throughput method for single-exosome analysis, to simultaneously profile more than a hundred surface proteins on a single exosome. PBA enables us to distinguish exosomes by highly heterogeneous surface protein compositions and identify exosome subpopulations in human plasma.

In this research, PBA was performed to detect a panel of 115 reported disease biomarkers at single-exosome resolution and subsequently classified all detected individual exosomes into 12 subpopulations according to their proteomic features. We examined the alteration of exosome subpopulations in primary CRC and liver-metastatic CRC groups compared to a healthy control (HC) group. A cancer-associated subpopulation increasing with CRC progression and an immune cells-associated subpopulation decreasing with CRC progression were identified, quantified, and profiled for proteomic features. The potential application of exosomal biomarkers as liquid biopsy targets was evaluated via a receiver operating characteristic (ROC) curve and then confirmed in an independent validation cohort. The identified critical exosome subpopulations were investigated for their ability in influencing the progression of CRC both in CRC cell lines in vitro and CRC mice experimental models in vivo. Thus, therapeutic interference of these exosome subpopulations provides an attractive approach for CRC treatment.

## Results

### Exosome proteomic biomarker analysis on single exosomes by PBA

**Fig. 1.**
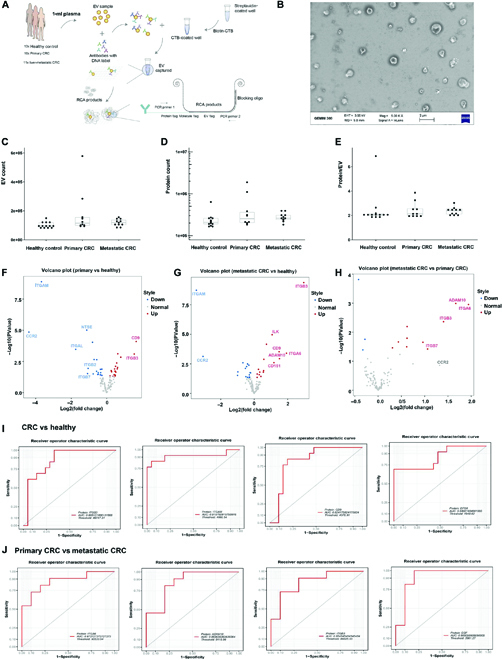
Proximity barcoding assay (PBA) for exosome proteomic biomarker analysis on single exosomes from the plasma samples of both healthy doners and CRC patients. (A) The working scheme of PBA. (B) The SEM picture of exosomes captured on the surface for PBA analysis. (C) The number of detected exosomes for each group of plasma samples. (D) The number of detected proteins for each group of plasma samples. (E) Detected proteins per exosome for each group of plasma samples. (F) Differential protein expression between primary CRC and HC group shown in the volcano plot. (G) Differential protein expression between live-metastatic CRC and HC group shown in the volcano plot. (H) Differential protein expression between live-metastatic CRC and primary CRC group shown in the volcano plot. (I) Receiver operating characteristic (ROC) curve of exosomal diagnostic biomarkers in CRC screening from HC. (J) ROC curve of prognostic biomarkers in distinguishing primary and metastatic CRC. Statistical analyses were performed using the paired t test. Each experiment was repeated at least 3 times.

The plasma donors enrolled in our study include patients with localized primary CRC without metastasis (*n* = 10), liver-metastatic CRC (*n* = 11), and healthy donors without diagnosed malignant disease (HC, *n* =13). Patients’ information was shown in Tables S1–S3 in supplementary information (SI). After plasma collection and exosome purification, PBA assay was performed in order to analyze the protein expressed on exosomes at single-exosome resolution. There were 115 biomarkers under investigation, which were previously reported in literatures, especially in cancer study (listed in SI [Table S4, detected proteins in PBA]). Figure 1A shows a scheme of the workflow. The exosomes are first captured onto a streptavidin-coated surface via biotinylated cholera toxin subunit B (biotin-CTB) through the interaction between CTB and ganglioside GM1 in lipid rafts of the vesicle membrane [18]. The captured exosomes are observed via scanning electron microscopy (SEM) in Fig. 1B. Then, the antibodies with conjugated oligonucleotides interact with proteins on exosomes and therefore were brought into proximity on the same exosome. Subsequently, the oligonucleotides in proximity obtain the same unique exosome barcoding tag during extension reaction. After library construction and DNA sequencing, the DNA sequences were used for extraction of antibody label tags, exosome barcoding tags, and molecule tags (i.e., unique molecular identifiers). Therefore, the protein expression for single exosomes was detected in each sample.

From 2 μ l of plasma samples, exosomes captured on the plate were detected via PBA. EVs are bound to the plate via affinity capture. The median number of exosomes detected via PBA, counted via EV tags, was 1.06 × 10^5^ per sample (Fig. 1C), while the median number of detected proteins, counted via molecule tags, were 2.42 × 10^5^ (Fig. 1D). No marked difference between HC group and CRC groups were observed. On average, 2.4 proteins were detected on each exosome (Fig. 1E). After trimmed mean of M-values (TMM) normalization, the differential expression of detected protein was shown in volcano plots (Fig. 1F to H) and dot plots (S5, protein expression-tmm in SI). Compared to the HC group, the exosome-associated expression of epidermal growth factor receptor pathway substrate 8, ITGB3, ITGA6, ADAM metallopeptidase domain 10 (ADAM10), integrin linked kinase, CD9, CD151, and ribosomal oxygenase 2 increased in plasma samples from CRC patients. Furthermore, the elevation of ITGA6, ITGB3, and ADAM10 was correlated with the metastasis of CRC. We also identified proteins that were significantly decreased in CRC groups, including ITGAM, 5'-nucleotidase ecto (NT5E), C-C motif chemokine receptor 2 (CCR2), Thy-1 cell surface antigen (THY1), Integrin alpha L (ITGAL), TIMP metallopeptidase inhibitor 1 (TIMP1), and matrix netallopeptidase 2 (MMP2). The data and statistic analysis of protein expression are shown in S6. differential expression of proteins in SI. These results show that proteomic features of exosomes were altered during CRC development.

To further evaluate the diagnostic biomarkers, ROC curves were plotted in Fig. 1I and J for selected biomarkers. In detail, to distinguish the CRC group from the HC group, the area under the curve (AUC) was 0.9158 (95% confidence interval [CI], 0.7842 to 1) for ITGAM, 0.8681 (95% CI, 0.7448 to 0.9915) for ITGB3, 0.8498 (95% CI, 0.7064 to 0.9932) for EPS8, and 0.8242 (95% CI, 0.6753 to 0.9731) for CD9. Meanwhile, for the prognostic biomarkers to discriminate primary CRC and metastatic CRC, the AUC was 0.9091 (95% CI, 0.7602 to 1) for epithelial growth factor, 0.8727 (95% CI, 0.7177 to 1) for ITGA6, 0.8636 (95% CI, 0.7012 to 1) for ADAM10, and 0.8545 (95% CI, 0.6878 to 1) for ITGB3, respectively.

### Exosome subpopulation alteration in CRC patients

**Fig. 2.**
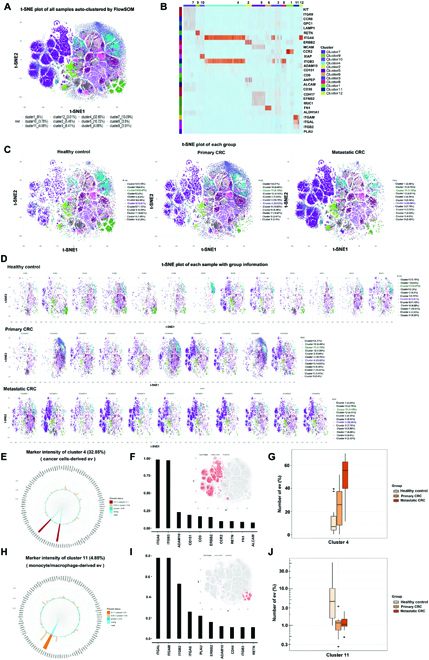
Exosome subpopulation alteration in CRC patients. (A) Exosome subpopulations from plasma samples of both CRC patients and healthy doners were determined via FlowSOM algorithm, an unsupervised machine learning process. Twelve subpopulations were shown in t-distributed stochastic neighbor embedding (t-SNE) plot. (B) proteomic biomarkers of each subpopulation were shown in heatmap. (C) The distribution of exosomes among each subpopulation were displayed for each sample groups, including HC, primary CRC and live-metastatic CRC group. (D) t-SNE plot for each sample with group information. (E to G) Cluster 4 featured expression of ITGA6 and ITGB3 increased significantly in CRC groups. (H to J) Cluster 11 with biomarkers of ITGAM, ITGAL, and ITGB2 were shown to decrease in CRC groups. Statistical analyses were performed using the t test.

To classify exosomes on the basis of the proteomic characteristics, an unsupervised machine learning algorithm named FlowSOM was applied to generate exosome clusters through a self-organizing map [19]. Exosome subpopulations were then visualized in t-distributed stochastic neighbor embedding (t-SNE) plot. PBA tests with an average of 1.06 × 10^5^ detected exosomes per sample were downsampled to 3,000 exosomes per sample for t-SNE visualization. The clustering of exosomes from all samples is shown in the t-SNE plot in Fig. 2A, in which 12 clusters were identified via FlowSOM. The biomarkers of each cluster were shown in Fig. 2B and S7.

Next, we analyzed the substantial phenotypic similarity and differences between CRC and HC groups. The distribution of exosomes among subpopulations were quantified and shown in a t-SNE plot for nontumor, primary CRC, and metastatic CRC groups (Fig. 2C). Comprehensively, exosome subpopulations for individual patient sample were displayed in Fig. 2D, and the statistical analysis of quantification were shown in SI (S8).

Among all subpopulations, the proportion of cluster 4 in total exosomes occupied the largest population (32.85% in Fig. 2A) and showed an increasing trend with the higher stage of CRC from 13.61% in the HC group to 23.03% in primary CRC and 52.06% in metastatic CRC (Fig. 2C, D, and G). The proteomic profiles of cluster 4 are shown in Fig. 2E, from which we could compare the expression frequency of each detected protein. For relatively highly expressed biomarkers, their expression levels were displayed in Fig. 2F, e.g., ITGA6, ITGB3, ADAM10, CD151, CD9 and ERBB2, where cluster 4 were color labeled in a t-SNE plot. Both ITGA6 and ITGB3 are dominantly expressed in cluster 4, and considering that they are documented as specific cancer markers [20,21], it implied that cluster 4 originates from cancer cells. Hereafter, we define cluster 4 as ITGA6/ITGB3 subpopulation.

On the other hand, cluster 11 possessed a comparatively low population (4.58% in Fig. 2A) and significantly decreased in the plasma samples of CRC patients, from 13.47% in the HC group to 1.13% and 1.13% in the primary CRC and metastatic CRC groups (Fig. 2C, D, and J). After featuring the protein expression in cluster 11, we found that ITGAM, ITGAL, and ITGB2 were relatively highly expressed (Fig. 2H and I). Given that ITGAM and ITGAL are reported as specific markers for monocyte/macrophages [22,23], our results inferred that cluster 11 originates from monocyte/macrophages. Hereafter, we define cluster 11 as ITGAM/ITGAL subpopulation.

### Malignancy of CRC cell lines were inhibited by plasma exosomes of healthy donors but promoted by that of liver-metastatic CRC patients

**Fig. 3.**
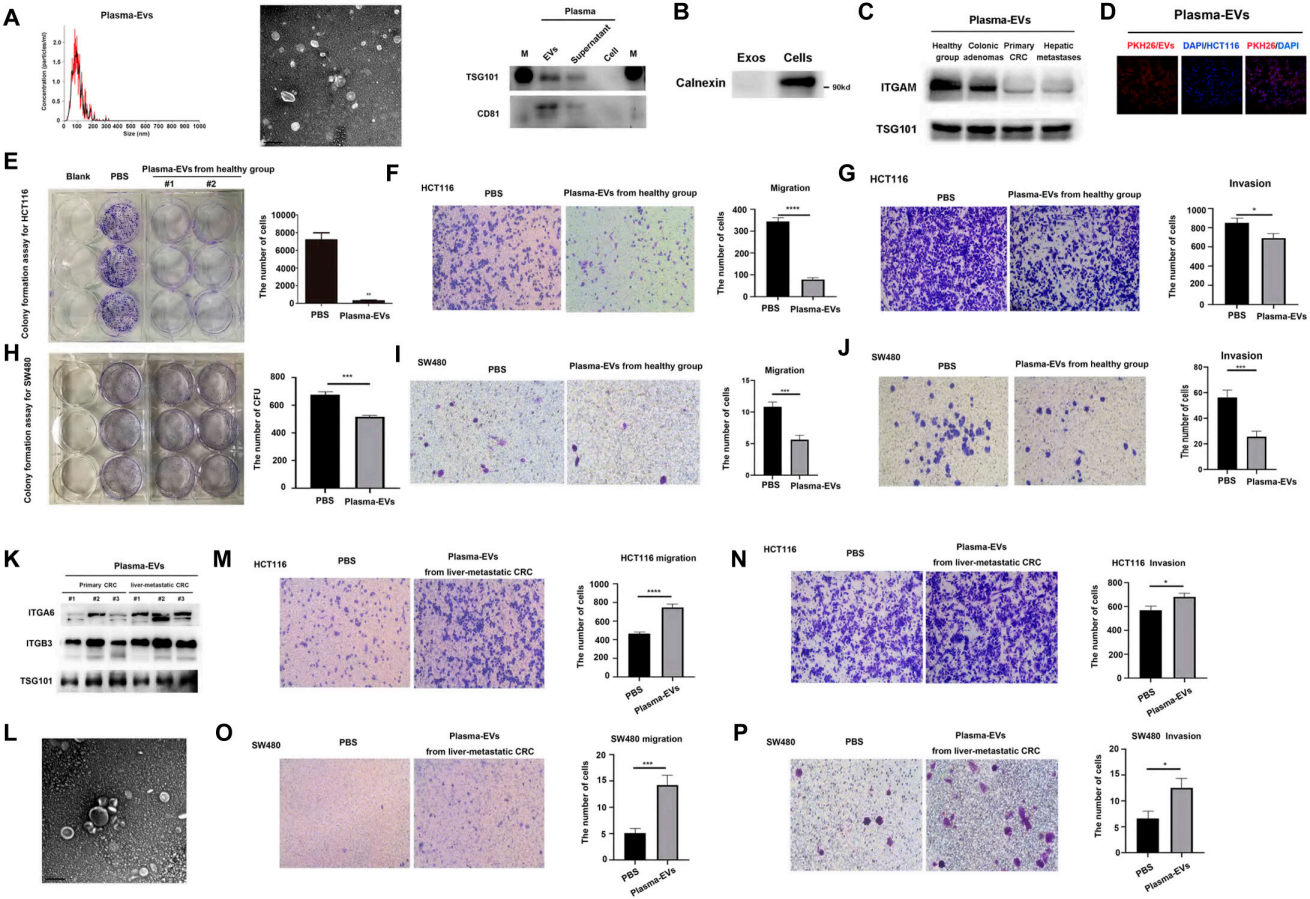
Plasma exosomes from healthy donors inhibit the migration and invasion of cancer cells, but plasma exosomes from liver-metastatic CRC patients have the opposite effect. (A) NTA and TEM image of plasma exosomes. Immunoblot of TSG101 and CD81 expression in plasma exosomes or exosomes supernatant from healthy donors, in which whole-cell lysates were used as a negative control. (B) Immunoblot of ITGAM expression in plasma exosomes from healthy group and primary CRC, where TSG101 was used as a loading control. (C) Immunoblot of ITGAM expression in plasma exosomes from healthy group, colonic adenomas and primary CRC, in which TSG101 was used as a loading control. (D) Immunofluorescence analysis of cellular uptake assays of exosomes. PKH26 (red) was used to stain exosomes, and DAPI (blue) was used to stain nuclei. (E to G) Representative images of colony formation assay, migration assay and invasion assay of HCT116 treated with PBS or plasma exosomes from healthy group and quantification. (H to J) Representative images of colony formation assay, migration assay and invasion assay of SW480 treated with PBS or plasma exosomes from healthy group and quantification. (K) Immunoblot of ITGA6 and ITGB3 expression in plasma exosomes from primary and liver-metastatic CRC patients. (L) Transmission electron microscopic image of plasma exosomes from liver-metastatic CRC patients. (M and N) Representative images of migration assay and invasion assay of HCT116 treated with PBS or plasma exosomes from liver-metastatic CRC patients and quantification. (O and P) Representative images of migration assay and invasion assay of SW480 treated with PBS or plasma exosomes from liver-metastatic CRC patients and quantification. Statistical analyses were performed using the paired t test. Data were calculated as means ± SD from at least 3 independent experiments. *P < 0.05; ***P < 0.001; ****P < 0.0001.

Then, we investigated the function of HC- and CRC-derived exosome in vitro. First, we extracted plasma exosomes from CRC patients and healthy individuals via ultracentrifugation and subsequently characterized the exosomes by Western blot, transmission electron microscopy (TEM) and nanoparticle tracking analysis (NTA) (Fig. 3A). NTA analysis showed plasma exosome had an average diameter of about 90 nm (Fig. 3A left panel). In addition, under TEM plasma exosomes of both HC and CRC groups presented as elliptical or round, double-layer-membrane vesicles (Fig. 3A middle panel and L). Moreover, in the Western blot, we also included the proteins extracted from supernatant of the ultracentrifugation step and from an HCT116 cell pellet as comparison. As shown in Fig. 3A right panel, both CD81 and TSG101, the exosomal biomarkers, were detected in the protein extraction of plasma exosomes but barely expressed in the protein extractions of the supernatant or cells. Figure 3B shows that calnexin was expressed in cells but not in exosomes. Subsequently, we examined the ITGAM expression in plasma exosomes from HC, colonic adenomas, primary CRC, and CRC with hepatic metastases. ITGAM was expressed the highest in HC, followed by colonic adenomas, and the lowest in primary CRC and CRC with hepatic metastases (Fig. 3C). This correlates with our previous results from the PBA analysis (Fig. 1F). In addition to samples from CRC patients, samples from the precursor adenomatous polyp-bearing patients (patients’ information in S9) had a lower expression of ITGAM as well, which collectively suggested that ITGAM-positive exosomes could be utilized as biomarkers for early diagnosis of CRC (Fig. 3C).

To further validate the cargo-transporting function of exosomes, we performed cellular uptake assays to verify that plasma exosome could be taken up by cultured CRC cell. Colocalization of PKH26 stained exosomes and 4',6-diamidino-2-phenylindole (DAPI)-stained HCT116 cells were observed under laser scanning fluorescent microscopy (Fig. 3D). Moreover, treatment with healthy human-derived plasma exosomes inhibited the proliferation (Fig. 3E and H and S10), migration (Fig. 3F and I), and invasion (Fig. 3G and J) of HCT116 and SW480; however, it had no impact on apoptosis of CRC cells (S11), indicating that exosomes from HC groups with ITGAM high expression failed to support malignancy of CRC cell lines.

Next, we extracted exosomes from plasma of CRC patients with liver metastasis and compared them to exosomes from primary CRC patients. The expression of ITGA6 and ITGB3 in plasma exosomes from liver-metastatic CRC patients was higher than that from primary CRC patients (Fig. 3K), which was consistent with our previous results of PBA (Fig. 1F). Furthermore, we analyzed the effects of liver-metastatic CRC patients-derived plasma exosomes on the invasion and migration of CRC cell lines including HCT116 and SW480. It showed that metastatic CRC-derived plasma exosomes significantly promoted migration (Fig. 3M and O) and invasion (Fig. 3N and P) of CRC cell lines. In conclusion, the in vitro studies performed on CRC cell lines suggest that healthy-donor-derived plasma exosomes with ITGAM-enhanced expression suppressed migration, invasion, and proliferation of the CRC cell line, while liver-metastatic CRC patients-derived plasma exosomes had the opposite effect and promoted the malignancy.

### Plasma exosomes from healthy donors suppressed growth of subcutaneous CRC tumor in mouse models, while plasma exosomes of liver-metastatic CRC patients promoted metastasis of CRC cells in vivo

**Fig. 4.**
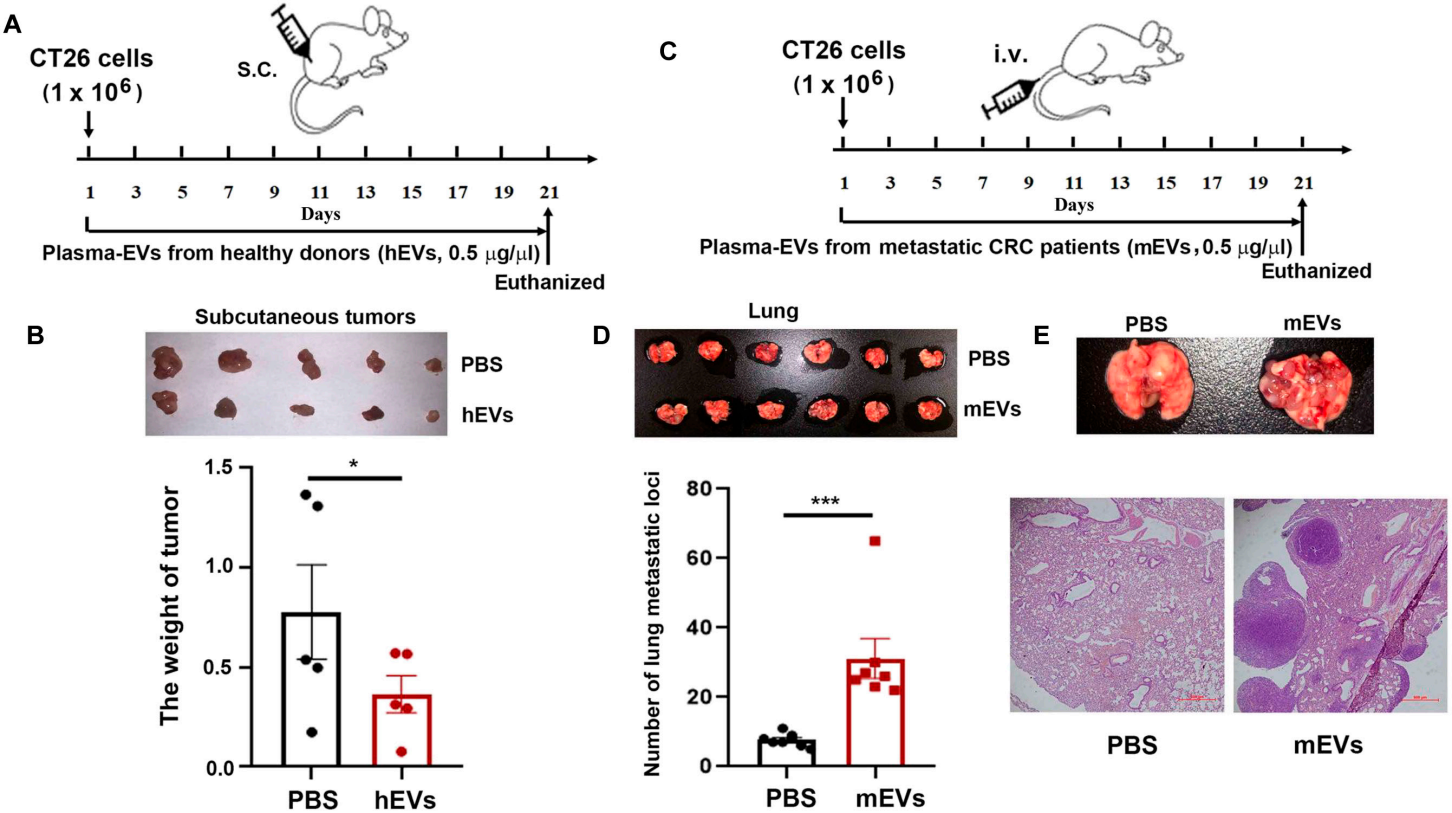
Plasma exosomes from healthy subjects inhibited the growth of subcutaneous tumor, but liver-metastatic CRC patients-derived plasma exosomes promoted migration and invasion of tumor cells. (A) Schematic diagram of tumor-bearing mice treatment. (B) Images of CT26 tumors at day 21 and histogram demonstrated the tumor weight per mouse. (C) Schematic diagram of lung metastasis tumor mice treatment. (D) Images of CT26 lung metastasis tumors in lung at day 21 with a histogram demonstrating the numbers of metastasis tumor in lung per mouse. (E) Representative images of hematoxylin and eosin stained of the lung metastasis tumor in indicated mice. Scale bar, 100 μm. Statistical analyses were performed using the paired t test. Each experiment was repeated at least 3 times. *P < 0.05; ***P < 0.001.

After revealing their tumor-suppressive function of plasma exosomes of healthy donors in vitro, we further performed subcutaneous implantation of CRC tumors in mice model to explore its therapeutic potential in vivo. Mouse CT26 CRC cells (1 × 10^6^) were injected subcutaneously into the right flank of each mouse at day 1 for both treatment group and control group. Healthy donor-derived plasma exosomes (hEVs) were injected at 50-μg protein per dose into the tail vein of each mice every other day from the day 1 to day 21 as the treatment group (Fig. 4A). For the control group, phosphate-buffered saline (PBS) was injected in the same procedure. Compared to the control group, tumor growth was decreased in treatment group with healthy-donor exosome injection, indicated by 43.4% decrease in tumor weight (Fig. 4B).

A CRC-metastasis mice model was constructed via intravenous injection of CT26 cells (1 × 10^6^ cells in PBS) to verify the tumor-promoting effect of plasma exosomes derived from patients with metastatic CRC. CT26 cancer cell-bearing mice were treated with liver-metastatic CRC patients-derived plasma exosomes (mEVs) at 50-μg protein weight per dose for each mouse every other day from day 1 to day 21 (Fig. 4C). The average number of lung metastatic foci was about 32.9 in mice treated with mEVs, while the control group with PBS injection instead has 7.6 metastatic foci on average (Fig. 4D and E). Histological analysis additionally validated the metastasis-promoting effect by plasma exosomes from liver-metastatic CRC patients.

Therefore, these studies demonstrated that healthy-donor-derived plasma exosomes inhibited the proliferation of subcutaneous tumor in vivo*.* Our data also proved plasma exosomes from CRC patients with liver metastasis promoted metastasis of CRC in vivo.

### ITGB3-rich exosomes promote invasion and migration of CRC cell line

**Fig. 5.**
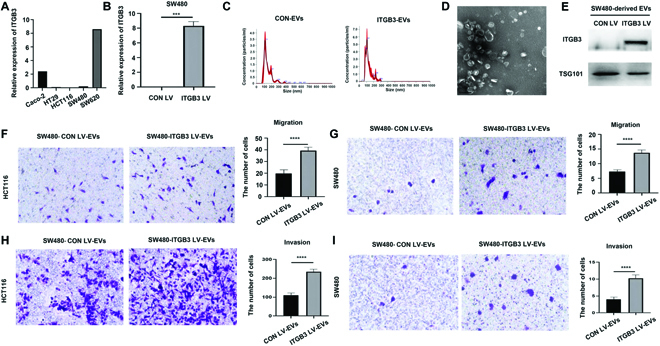
ITGB3-rich exosomes promote migration and invasion of colon cancer cell lines. (A) Quantitative RT-PCR analysis of ITGB3 expression in Caco-2, HT29, HCT116, SW480, and SW620 cell lines. Relative mRNA levels were determined by normalization to the housekeeping gene GAPDH. (B) Quantitative RT-PCR analysis of ITGB3 expression in SW480 with (ITGB3 LV) or without (CON335), where data of CON335 served as a control after normalization to GAPDH. (C to E) NTA analysis, transmission electron microscopic image and immunoblot of ITGB3 expression of SW480-derived exosomes from (B). (F and H) Representative images of migration assay and invasion assay of HCT116 treated with SW480-derived exosomes from (B) with quantification. (G and I) Representative images of migration assay and invasion assay of SW480 treated with SW480-derived exosomes from (B) and quantifications. Statistical analyses were performed using the paired t test. Data were calculated as means ± SD from at least 3 independent experiments. ***P < 0.001; ****P < 0.0001.

We further evaluated the function of ITGB3-enriched exosomes in vitro due to the importance of ITGB3 in cancer biology and its representative role in altered exosome subpopulation in CRC groups according to PBA tests (Fig. 1E and F). First, we detected the expression level of ITGB3 through real-time quantitative polymerase chain reaction (RT-qPCR) in several CRC cell lines including SW620, SW480, HCT116, HT29, and Caco-2, in which Caco-2 and SW620 showed relatively high expression of ITGB3 (Fig. 5A). ITGB3 is expressed at a low level in HCT116 and HT29. Expression of ITGB3 in SW620 increased by about 8 times compared to SW480 (Fig. 5A). Interestingly, SW480 was collected from a primary tumor in the colon, while SW620 was collected from a metastatic site in a lymph node from the same patient 1 year later. Hereafter, we overexpressed ITGB3 in SW480 cells via lentiviral transfection (SW480-ITGB3 LV cells). The mRNA level of ITGB3 was examined in RT-qPCR, in which significant upregulation of ITGB3 was confirmed in SW480-ITGB3 LV cells compared to its control cell line SW480-CON LV, i.e., SW480 with control lentiviral transfection (Fig. 5B). Subsequently, we extracted exosomes from SW480-CON LV and SW480-ITGB3 LV cell cultures and characterized exosomes with NTA (Fig. 5C) and TEM (Fig. 5D). The Western blot showed ITGB3 expression was increased in exosomes of SW480-ITGB3 LV cells (Fig. 5E). Furthermore, we investigated the impacts of ITGB3-rich exosomes on migration and invasion capability of CRC cell lines. HCT116 and SW480 cells were treated with exosomes secreted by ITGB3 overexpressing SW480-ITGB3 LV cells and its control cell line SW480-CON LV, respectively. It showed that cell migration (Fig. 5F and G) and invasion ability (Fig. 5H and I) were significantly promoted in ITGB3-rich exosomes-treated group. In summary, upregulation of ITGB3 expression in SW480 cells leads to elevation of ITGB3 level in secreted exosomes, which could promote migration and invasion of CRC cell lines including HCT116 and SW480.

### ITGAM-rich exosomes secreted by M1 macrophages inhibit malignancy of CRC cell lines, tested in vitro

**Fig. 6.**
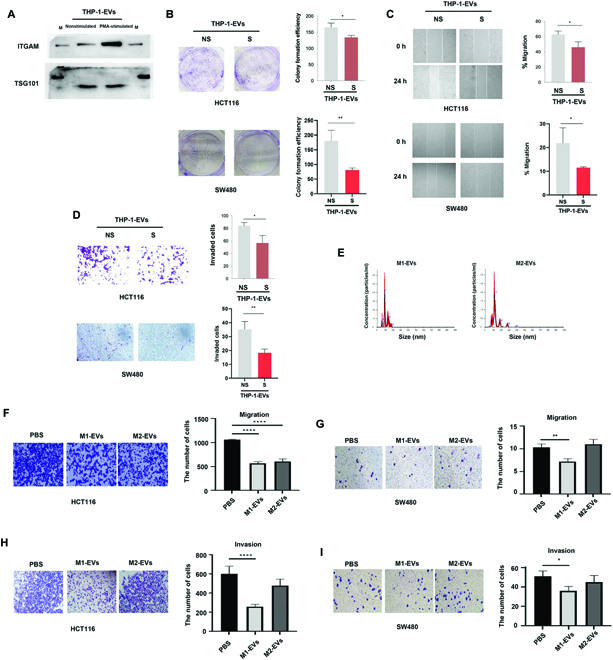
ITGAM-rich exosomes secreted by M1-like macrophages inhibit the ability of colon cancer cell lines to proliferate, migrate, and invade. (A) Immunoblot of ITGAM expression in exosomes from THP-1 treated with or without PMA. (B to D) Representative images of colony formation assay, migration assay, and invasion assay of HCT116 treated with exosomes from THP-1 with (S) or without (NS) PMA-stimulated and quantifications. (E) NTA analysis of exosomes from M1-like macrophages and M2-like macrophages. (F and H) Representative images of migration assay and invasion assay of HCT116 treated with exosomes from indicated groups with quantifications. (G and I) Representative images of migration assay and invasion assay of SW480 treated with exosomes from indicated groups with quantifications. Statistical analyses were performed using the paired t test. Data were calculated as means ± SD from at least 3 independent experiments. *P < 0.05; **P < 0.01; ***P < 0.001; ****P < 0.0001.

To further investigate the ITGAM-rich exosomes secretion from macrophages and their role in cancer progression, we cultured a human monocytic THP-1 cell line and stimulated THP-1 cells with phorbol-12-myristate-13-acetate (PMA) to obtain differentiated macrophages [24]. We collected exosomes from both THP-1 and PMA-stimulated THP-1 cells (PMA/THP-1). As shown in Western blots, we demonstrated that PMA/THP-1 cells have higher ITGAM expression in exosomes than THP-1 cells (Fig. 6A). Compared to THP-1 exosomes, PMA/THP-1 exosomes significantly inhibited cell proliferation, migration, and invasion of HCT116 and SW480 cell lines (Fig. 6, B to D).

In order to determine the cancer-suppressive effect of exosomes generated by specific types of macrophages, we extracted bone marrow-derived macrophages (BMDMs) from mice for exosome production. Through stimulation of BMDM with lipopolysaccharide (LPS) and interleukin-4 (IL-4), we then successfully obtained M1-like macrophages and M2-like macrophages, respectively. We collected exosomes from supernate of M1 and M2 macrophages. Then, we performed NTA to quantify extracted exosomes (Fig. 6E). Our data showed that both M1- and M2-originated exosomes suppress the migration of the HCT116 cell line (Fig. 6F). We demonstrated exosomes secreted by M1-like, not M2-like, macrophages significantly inhibited migration in SW480 (Fig. 6G) and invasion (Fig. 6H and I) in both HCT116 and SW480 cells. These results revealed that M1 macrophage-derived ITGAM-rich exosomes played suppressive roles in the invasion and migration of CRC cell lines.

### ITGAM-rich exosomes inhibited the proliferation of subcutaneous CRC tumor in mouse models; however, ITGB3-rich exosomes promoted metastasis of CRC cells in vivo

**Fig. 7.**
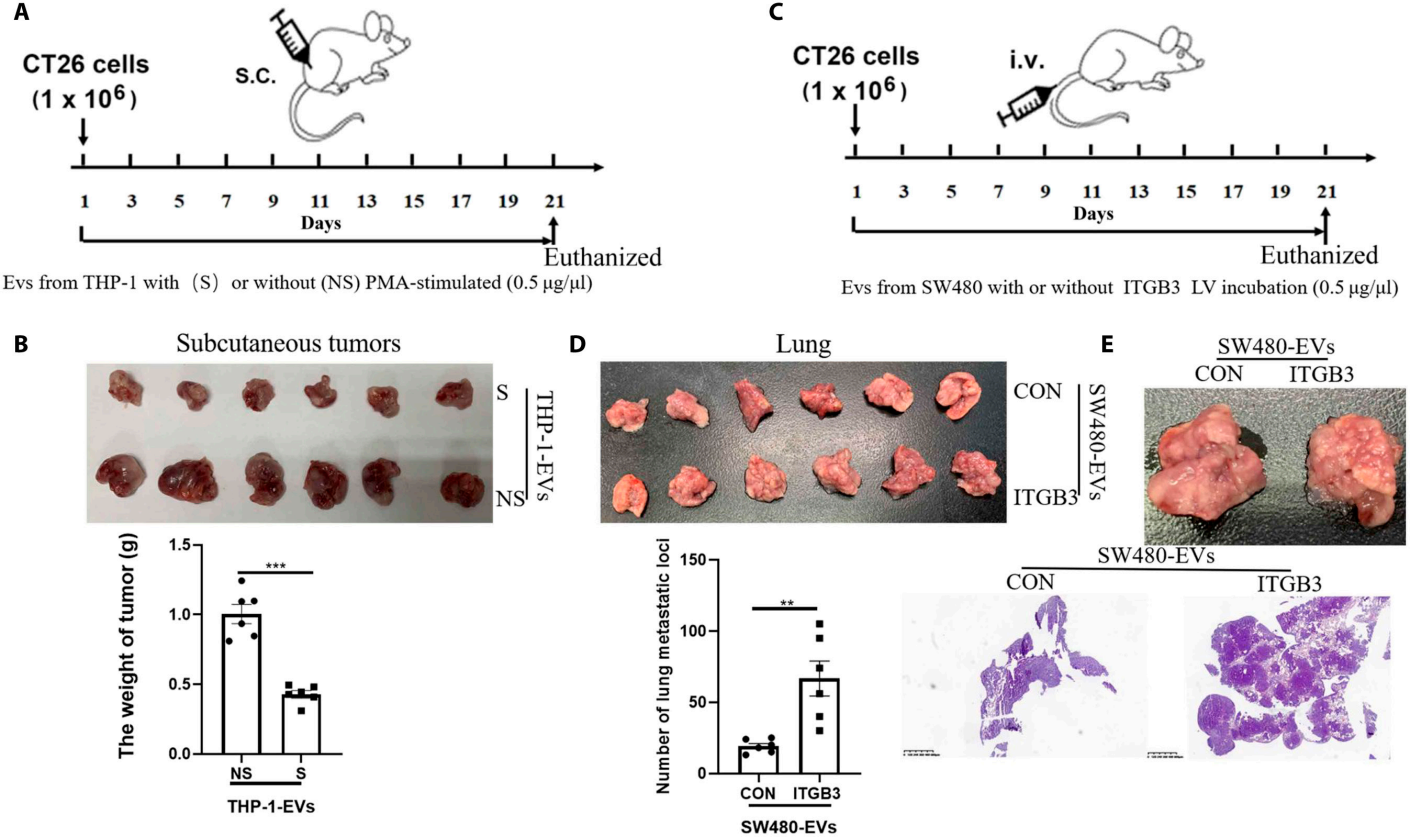
ITGAM-rich exosomes inhibited the growth of subcutaneous CRC tumor in mice models, while ITGB3-rich exosomes promoted metastasis of CRC cells in vivo. (A) Schematic diagram of tumor-bearing mice treatment. (B) Images of CT26 tumors at day 21 and histogram demonstrated the tumor weight per mouse. (C) Schematic diagram of lung metastasis tumor mice treatment. (D) Images of CT26 lung metastasis tumors in lung at day 21 with a histogram demonstrating the numbers of metastasis tumor in lung per mouse. (E) Representative images of hematoxylin and eosin stained of the lung metastasis tumor in indicated mice. Scale bar, 100 μm. Statistical analyses were performed using the paired t test. Each experiment was repeated at least 3 times. *P < 0.05; ***P < 0.001.

CRC tumors subcutaneous implantation in mice model was used to explore therapeutic potential of ITGAM-rich exosomes in vivo. Mouse CT26 CRC cells (1 × 10^6^) were injected subcutaneously into the right flank of each mouse at day 1 for both treatment group and control group. PMA/THP-1 exosomes were injected at 50 μg protein per dose into the tail vein of each mice every other day from the day 1 to day 21 as the treatment group (Fig. 7A). For the control group, THP-1 exosomes were injected in the same procedure. Compared with the control group, decreased tumor growth was observed in the treatment group with PMA/THP-1 exosome injection, indicated by 57.4% decrease in tumor weight (Fig. 7B).

A CRC-metastasis mice model was constructed via intravenous injection of CT26 cells (1 × 10^6^ cells in PBS) to verify the tumor-promoting effect of ITGB3-rich exosomes. CT26 cancer cell-bearing mice were treated with exosomes secreted by ITGB3 overexpressing SW480-ITGB3 LV cells and its control cell line SW480-CON LV at 50-μg protein weight per dose for each mouse every other day from day 1 to day 21 (Fig. 7C). The average number of lung metastatic foci was about 66.7 in mice treated with SW480-ITGB3 LV cell-derived exosomes, while the control group with SW480-CON LV cell-derived exosomes injection instead has 19.2 metastatic foci on average (Fig. 7D and E). Histological analysis additionally validated the metastasis-promoting effect by ITGB3-rich exosomes.

These studies demonstrated that ITGAM-rich exosomes inhibited the proliferation of subcutaneous tumor in vivo. Our data also showed that ITGB3-rich exosomes promoted metastasis of CRC in vivo.

### PBA of external validation cohort confirms the diagnostic value of exosomal biomarkers in CRC

**Fig. 8.**
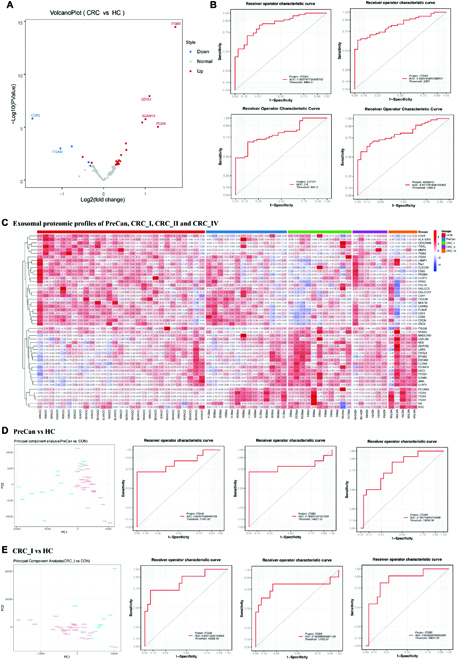
PBA for diagnosis of CRC in the validation cohort of 30 CRC patients and 58 HC, and exosome profiling of precancerous lesions and stage I CRC cohorts reveals the potential of exosomal biomarkers for early screening of CRC. (A) Differential protein expression between CRC and HC group shown in the volcano plot. (B) ROC curves of exosomal diagnostic biomarkers (ITGA6, ITGB3, CD151 and ADAM10) in CRC screening from HC. (C) the differential expression of 46 exosomal proteins between CRC groups and HCs was shown in heatmap. (D) ROC curves of diagnostic biomarkers in precancerous lesions of CRC screening from HC. (E) ROC curves of diagnostic biomarkers in distinguishing stage I CRC and HC.

To further validate the potential of exosomal biomarkers in the diagnosis and prognosis of CRC, we performed PBA to detect the biomarkers in an independent cohort of patients as external validation cohort (30 CRC patients and 58 HC) from Guangdong Province in southern China, whereas the discovery cohort was enrolled in Shandong Province in eastern China. As mentioned in Figs. 1 and 2, EV subpopulations with expression of ITGA6, ITGB3, CD9, CD151, and ADAM10 showed significantly differential expression and were selected as potential diagnostic and prognostic biomarkers. In this validation test, the AUC was 0.834 for ITGA6 and 95% CI is (0.7529, 0.9155). The AUC was 0.856 for ITGB3 and 95% CI is (0.7779, 0.9336). The AUC was 0.812 for ADAM10 and 95% CI is (0.7247, 0.8989). The AUC was 0.8 for CD151 and 95% CI is (0.7092, 0.8908) (Fig. 8). Additionally, we also observed the elevated ratio of ITGA6-ITGB3 subpopulation, decreased ratio of CCR2 subpopulation, and ITGAM-ITGAL-ITGB2 subpopulation in CRC groups, as shown in S12 and S13, which correlated with our findings in Figs. 1 and 2.

### Exosome profiling of precancerous lesions and stage I CRC cohorts reveals the potential of exosomal biomarkers for early screening of CRC

To further verify the potential of exosomal biomarkers in the early screening of CRC, we performed PBA to profile the plasma exosomes in precancerous lesions (PreCan) and stage I CRC (CRC_I) patients (14 PreCan patients, 11 CRC_I patients, and 28 HC) enrolled in Qilu Hospital of Shandong University in Shandong Province, China. The PBA analysis in this section utilized an upgraded panel with 200-plex antibodies for detection of mainly cellular adhesion molecules (panel information in S14).

As shown in the heatmap of Fig. 8C, after TMM normalization of protein expression data and analysis of variance, we obtained the differentially expressed exosomal proteins including 26 proteins with higher expression and 23 proteins with lower expression. Expression of proteins in ITGA6-ITGB3 subpopulation and ITGAM-ITGAL-ITGB2 subpopulation confirmed our findings in our CRC cohort and validation cohort. ITGA6, ITGB3, ITGAM, ITGAL, and ITGB2 showed significantly differential expression. To evaluate the diagnostic value of exosomal proteins as CRC screening biomarkers, we analyze the differentially expressed proteins with ROC and calculate the AUC values. In Fig. 8D, we showed results for distinguishing PreCan group from the HC group. The AUC was 0.835 (95% CI, 0.678 to 0.992) for ITGA6, 0.783 (95% CI, 0.582 to 0.985) for ITGB3, and 0.786 (95% CI, 0.642 to 0.930) for ITGAM. In Fig. 8E, we showed ROC analysis for distinguishing the CRC_I group from the HC group. The AUC was 0.840 (95% CI, 0.683 to 0.997) for ITGA6, 0.784 (95% CI, 0.562 to 1) for ITGB3, and 0.850 (95% CI, 0.709 to 0.990) for ITGB2.

## Discussion

The exosome heterogeneity can provide plentiful and valuable information that cannot be easily obtained by traditional exosome analysis techniques. In a body fluid sample, the disease-associated exosomes can be very low in abundance compared to other exosomes, which obscures their diagnostic potential. We investigated the surface protein profile of single exosomes using PBA, a novel method for exosome analysis. We succeeded in identifying a large quantity of exosomes directly from human plasma and to profile the combination of proteins on individual exosomes. This study provides plentiful of not only clinical but also experimental evidence that cancer cell-derived ITGB3+ exosomes mediated CRC progression and metastasis and macrophage-derived ITGAM+ exosomes mediated CRC suppression.

Integrins facilitate adhesions between cell and microenvironment and mediate signal communication through the downstream pathways. The aberrant expression and distribution of ITGB3 is reported to facilatate cancer metastasis [25–27]. It was reported that an ITGB3 antagonist could inhibit CRC metastasis in a mouse model [28]. ROS could significantly upregulated ITGB3 expression and promoted invasiveness and increased migration and invasion in CRC cell lines [29]. Moreover, it was reported that miR-30a-5p could suppress the expression of ITGB3 and thus inhibited CRC metastasis [30]. In our study, we detected exosomes at single-particle resolution to reveal the existence of an exosome subpopulation featured expression of ITGB3, ITGA6, CD151, CD9, and ERBB2. The proportion of this subpopulation in total plasma exosomes increases with development of CRC, which makes it a possible target for CRC screening, diagnosis, and prognosis. We confirmed the cancer-promoting role of ITGB3 and proved the correlation of ITGB3+ exosomes with CRC progression and metastasis in vitro and in vivo. Besides diagnostic and prognostic value of ITGB3+ exosome subpopulation, targeting ITGB3 may also serve as a hopful therapeutic target in CRC management.

Both KRAS-wild type and KRAS-mutant cell lines were used for in vitro and in vivo experiments. Our results could still pose a research question of whether the function of ITGB3- and ITGAM-positive exosomes is associated with RAS signaling. There is no literature that relates ITGB3 and ITGAM to Kras so far. Thus, whether these results are subtype nonspecific needs to be verified by further validation.

Crosstalk between CRC and infiltrating macrophage reeducates macrophage to form the complex tumor microenvironment [31]. Recent studies have pointed out exosome-mediated intracellular communication [32], in which exosomes can be absorbed by local tissue or reach the target organs and precondition distant target organs through endocytosis [33,34]. Depending on the phenotype, macrophages are a double-edge sword in cancer development. M1 macrophage is proinflammatory by delivering Th1 immune responses to inhibit tumorigenesis by secreting of IFN-γ and IL-1. On the other side of the coin, M2 macrophage functions through secreting IL-8 and IL-4, to form an immune-suppressive microenvironment to facilitate tumor development [35]. Macrophages influence CRC cell behaviors by secreting many soluble molecules [36,37]. Our observation is a potent evidence to support that macrophage-derived exosomes deliver a cancer-suppressive role and inhibit the progression of CRC. ITGAM+ exosome subpopulation derived from macrophage could serve as a biomarker for screening and early diagnosis of CRC.

With the aid of PBA method, we were able to profile up to 200,000 individual exosomes for each sample and therefore screen CRC biomarkers at the single-exosome level. Like single cell analysis, we could perform single-exosome clustering via machine learning method and identify exosome subpopulations. We could observe the alteration of exosome subpopulations during CRC progression. The disadvantage is that the detection panel is based on selected antibodies, and it is beneficial if more protein types could be invesitgated after expansion of the PBA detection panel. So far, our study contain 3 independent validation cohorts. In the future, we continue to expand the sample size to validate the value of the ITGB3+ and ITGAM+ exsome subpopulations in early CRC screening and early detection of CRC metastasis.

In summary, our study uses a novel method to analyze individual exosomes and displays different exosome subpopulation patterns along CRC development. We provide compelling evidence that cancer cell-derived ITGA6-ITGB3-positive exosomes facilitate tumor proliferation and metastasis; however, macrophage-derived ITGAM-positive exosomes inhibit CRC development and metastasis. They are promising tissue-specific biomarkers for liquid biopsy to predict early onset and metastasis of CRC. Moreover, therapeutic interference of these 2 exosome subpopulations provide an attractive approach for treating CRC.

## Methods

### Human blood samples and cell lines

The study was approved by the Medical Ethics Committee of Qilu Hospital of Shandong University (KYLL-202011-209-01). The study was conducted according to the principles expressed in the Declaration of Helsinki. From January 2019 to December 2020, a total of 10 patients with localized primary CRC and 11 patients with liver-metastatic CRC were enrolled in our study in Shandong University Qilu Hospital. Six patients with colon adenomas were enrolled from Qianfoshan Hospital of Shandong First Medical University. Thirteen healthy volunteers were enrolled as the HC group. All sample donors signed informed consent. The study of validation cohort were approved by the Ethical committee of Shenzhen Sencond People’s Hospital. A total of 30 CRC patients and 58 healthy donors were enrolled in the study from July 2019 to June 2020. Another validation cohort included 28 HC, 14 precancerous lesions of CRC patients, 11 stage I CRC patients, 6 stage II CRC patients, and 5 stage IV CRC patients. These patients are from Shandong University Qilu Hospital, from January 2022 to May 2022. All sample donors signed informed consent.

The intravenous blood was collected from each sample donor and stored in collective tubes with EDTA-K2 as anticoagulants. Plasma sample was prepared via whole-blood centrifugation at 3000 RPM for 15 min. Human monocytic THP-1 cell line, mouse CRC cell line CT26, and human CRC cell lines HCT116, Caco2, SW480, SW620, HT29 were all purchased from ATCC. HCT116, Caco2, SW480, HC29, SW620, and THP-1 were cultured with RPMI 1640 with addition of 10% fetal bovine serum (FBS) and 1% penicillin and streptomycin (PS). Exosome-depleted FBS was used instead of regular FBS for the exosome production. The exosome-depleted FBS was depleted of exosomes by ultracentrifuging at 100,000×*g* for 18 h at 4 °C [38].

### Isolation and culture of cells

The isolation and culture of mouse BMDMs were as described in literature [39]. In short, bone marrow was extracted from the femur of mice, and red blood cells were lysed. After 7 days of the bone marrow cells culture, M0 macrophages were obtained by adding Dulbecco's modified Eagle's medium supplemented with 40% L929 conditioned medium and 10% heat-inactivated FBS. For M1-like activation, 0.5 to 0.7 × 10^6^ macrophages were plated in tissue culture dishes and treated with LPS (100 ng/ml, Invivogen, America) for 1 d. For M2 polarization, 1 × 10^7^ macrophages were treated with IL-4 (20 ng/ml, Peprotech,America) for 1 d.

### Exosome purification and characterization

Exosomes were isolated by differential centrifugation. In short, 3-step centrifugation at 300 ×*g* for 10 min, 2000 ×*g* for 10 min, and 10,000 ×*g* for 30 min was performed to remove cells, possible apoptotic bodies, cell debris, or biomolecule aggregates from the plasma or cell culture medium. Then, the supernatants were centrifuged at 100,000 ×*g* for 70 min (Optima XPN Ultracentrifuge with 32Ti rotor, Beckman Coulter, Germany) for pelleting exosomes. After resuspending with PBS, exosomes were precipitated once more via ultracentrifugation at 100,000 ×*g* for 70 min. All purified exosomes were resuspended in PBS. The size distribution and particle concentration of exosome extractions were characterized by NTA (NanoSight NS300, Malvern Panalytical, UK). Protein markers CD81 (sc-7637, Santa Cruz, America) and TSG101 (sc-7964, Santa Cruz, America) of isolated exosomes were detected by Western blotting. The purified exosomes were imaged with a transmission electron microscope (TEM, JEOL JEM-1200EX, JEOL, Japan) with electron beam at a voltage of 80 KV.

### PBA

EV samples from patients’ plasma were analyzed with PBA according to the protocols in our former study [17]. A panel of 112 antibodies were conjugated with oligonucleotides containing unique protein tags, unique molecular tags, and also universal sequence for primer binding. Biomarkers were detected at the single-exosome level with PBA. Two microliters of plasma was mixed with 1 μL of PBA buffer and then incubated with 1 μl of antibody mix (2 μg/ml for each antibody) for 2 h at room temperature.

To prepare exosome capture plate, biotinylated cholera toxin subunit B (biotin-CTB, 2.5 μg/ml in PBS, C34779, Thermo-Fisher Scientific, USA) was added to each well of a 96-well plate with streptavidin coating (PCR0STF-SA5/100, Biomat, Italy). The plate was incubated for 20 min at room temperature and washed 3 times with PBS with Tween 20 (PBST). Plasma/antibody mix were diluted to a volume of 20 μl with PBA buffer and transferred into each well of CTB coated plate for affinity capture of exosomes through the interaction between CTB and GM1 enriched in lipid membrane of exosomes. Captured exosomes were fixed with paraformaldehyde solution (4%) and imaged with SEM (SU8010, Hitachi, Japan) utilizing 3-kV beam energy and a secondary electron detector.

The oligonucleotides on the same exosome obtain a unique exosome tag during the extension reaction after binding of an exosome tag templates. Sample index and sequencing adapters was added through designed primers in PCR. The library was sequenced with Illumina NextSeq (CN500, Illumina, USA) utilizing single-end 75-bp sequencing reagent kit (20024906, Illumina, USA). After DNA sequencing, data of each sample was obtained as bcl file. The PBA experiment were performed in SecreTech (Shenzhen, China) according to the standard operational procedure provided by Vesicode (Solna, Sweden).

### RNA extraction and real-time PCR analysis

EASY spin Plus kit (Aidlab, China) and QuantiTect Rev (Vazyme, China) were used to obtain RNA from tissues or cells. Transcription Kit (Vazyme, China) was used to synthesize QuantiTect RevComplementary DNA (cDNA), and cDNA was amplified by SYBR Green qPCR Mix (Vazyme, China) and analyzed by RT-PCR.

Primer list used in this study are as follows:

ITGB3 FORWARD:TCATCTGGAAACTCCTCATCAC

ITGB3 REVERSE:GTAGACGTGGCCTCTTTATACA

### Western blotting analysis

Total proteins from cells or exosomes were extracted with RIPA buffer (Beyotime, China) with addition of protease and phosphatase inhibitor cocktails (Selleck, China). The protein concentrations were quantified with the BCA kit (Beyotime, China), and Western blot was performed as described previously [40]. Detection and imaging was performed with Gel imager (Pinghao, Beijing), the chemiluminescent horseradish peroxidase substrate BrightTM ECL (Beyotime, China).

### Cell migration and invasion assay

Cell migration and invasion assays were performed in transwell tests. For cell migration test, 5 × 10^5^ cells in 200 μl of FBS-free RPMI 1640 was added to upper chamber of transwell inserts with diameter of 6.5 mm and pore size of 8 μm (Corning, USA). In the 24-well plate with transwell inserts, 600 μl of RPMI 1640 culture medium containing 10% FBS and 1% PS was added to the lower part of the chamber. For invasion tests, a Matrigel layer was prepared on the bottom of the upper chambers before cell seeding. After incubation at 37 °C, 5% CO_2_ for 24 h, the upper chamber was cleaned gently with cotton swabs, fixed with 4% paraformaldehyde, stained with crystal violet staining (Beyotime, China), and washed 3 times with water. The cells migrated to the other side of the filter was imaged using an inverted microscope (Nikon, Shanghai). The quantification of cells were performed by cell counting from 8 images of the same transwell test. Duplicate of transwell test were done for each condition. 10 μg/ml exosomes were used for functional assays.

### Colony formation

A total of 5 × 10^4^ treated cells were coated into 6-well plates with 3 repetitions. After 7-d incubation, these plates were washed with PBS twice, fixed by methanol for 10 min, and stained with 0.1% crystal violet solution within 10 min for further analysis. We use 10μg/ml for functional assays.

### Imaging of fluorescently labelled exosomes and cells

PKH26 (Sigma-Aldrich, USA) at concentration of 1 μM was used to label exosomes. The PKH26-labeled exosomes were incubated with SW480 cells for 20 h, and the cell nuclei were stained with DAPI (beyotime, Shanghai). The slides were imaged with a laser scanning confocal microscope Axio-Imager-LSM880 (ZEISS, Germany).

### Tumor mouse model

A subcutaneous tumor mouse model was prepared by subcutaneously inoculation of 1 × 10^6^ CT26 mouse colon cancer cells to 7-week-old BALB/c mice. Colon cancer lung metastasis model were prepared by injection of 1 × 10^6^ CT26 cells to the tail vein of 7-week-old BALB/c mice. For exosome injection of mice model, plasma exosomes from primary CRC or metastatic CRC patients were injected every other day from day 1 to day 21 through the tail vein. The same procedure of PBS injection was applied as control. The mice were euthanized at day 21 for examination of tumor development and metastasis.

### Cell lentivirus transfection assay

A recombinant lentivirus containing pUbi-MCS-3FLAG-CBh-gcGFP-IRES-puromycin was constructed on vector GV492 for transfection of cells according to the manufacturer's instructions. The control virus was CON335, and the overexpressed virus was ITGB3-LV. According to the virus titer in the instructions, a 6-well plate containing 1 × 10^6^ SW480 cells containing 10 μl per well was given to control virus CON335 and overexpressed virus ITGB3-LV, respectively.

### Data processing and statistical analysis

The bcl files from DNA fragment sequencing were converted to fastq file with bcl2fastq software (Illumina). With the aid of FASTX-Toolkit, the DNA sequences were filtered on the basis of sequencing quality, i.e., more than 75% base with Phred Quality Score of 20 or above. Data quality control was performed with FastQC, and clean data were used for further analysis. DNA sequences were deduplicated according to the molecule tag in the sequences to obtain unique reads. Unique exosome tags were extracted and recorded as identifiers for single exosomes. By mapping of protein tags in the sequences to the sequences of oligonucleotides conjugated to antibodies, the protein expression on each single exosome were counted. The file consists of ID of single exosomes and their protein expression profiles were obtained for each sample.

The protein expression dataset was obtained by summation of protein counts in all exosomes in each sample. The TMM normalization was performed for comparison of protein expression level. Differential expression analysis was performed in edgeR. Shapiro–Wilk test was applied to test the normality of datasets. Student *t* test was used for normally distributed dataset with equal variance and Welch’s test for normally distributed unequal variances. The false discovery rate (FDR) was controlled with the Benjamini and Hochberg approach. FDR-adjusted *P* values < 0.05 were considered significant.

For EV subpopulation analysis, FlowSOM, an unsupervised machine learning algorithm, and ConsensusClusterPlus was applied [19]. The clustering of EVs was visualized in t-SNE plot. The proteomic profiles of each EV subpopulation were plotted in heat map. The proportion of each subpopulation was quantified for each sample group.

## Data Availability

All data generated or analyzed during the current study are included in this published article (and its supplementary information files) or available on published datasets (TCGA or GEO).
