# Supplementary material for: Single-Exosome Profiling Identifies ITGB3+ and ITGAM+ Exosome Subpopulations as Promising Early Diagnostic Biomarkers and Therapeutic Targets for Colorectal Cancer
Source: Research (Wash D C). 2023 Jan 30;6:0041. doi: 10.34133/research.0041 (PMC10076010; doi:10.34133/research.0041)
Supplement: Supplementary 1 — Tables S1 to S15 [file research.0041.f1.pdf]

Table S1 Clinical characteristics of 11 CRC stage IV patients enrolled in our study.

Patient No.	Sex	Pathology	AJCC Stage	Liver metastasis	MSI status	Metabolic disorders
CRC stage IV patient 1	Female	Adenocarcinoma	IV	Yes	MSS	No
CRC stage IV patient 2	Female	Adenocarcinoma	IV	Yes	MSS	No
CRC stage IV patient 3	Male	Adenocarcinoma	IV	Yes	MSS	No
CRC stage IV patient 4	Female	Adenocarcinoma	IV	Yes	MSS	No
CRC stage IV patient 5	Male	Adenocarcinoma	IV	Yes	MSS	No
CRC stage IV patient 6	Male	Adenocarcinoma	IV	Yes	MSS	No
CRC stage IV patient 7	Female	Adenocarcinoma	IV	Yes	MSS	No
CRC stage IV patient 8	Male	Adenocarcinoma	IV	Yes	MSS	No
CRC stage IV patient 9	Male	Adenocarcinoma	IV	Yes	MSS	No
CRC stage IV patient 10	Male	Adenocarcinoma	IV	Yes	MSS	No
CRC stage IV patient 11	Male	Adenocarcinoma	IV	Yes	MSS	No

Abbreviation: AJCC, American Joint Committee on Cancer; MSI, microsatellite instability.

Table S2 Clinical characteristics of 10 CRC stage II patients enrolled in our study.

Patient No.	Sex	Pathology	Tumor Size(cm)	AJCC Stage	LN metastasis	Distant metastasis	MSI status	Metabolic disorders
CRC stage II patient 1	Female	Adenocarcinoma	4.0 x 0.5	II	No	No	MSS	No
CRC stage II patient 2	Male	Adenocarcinoma	3.5 x 1.8	II	No	No	MSS	No
CRC stage II patient 3	Male	Adenocarcinoma	7.0 x 1.0	II	No	No	MSS	No
CRC stage II patient 4	Male	Adenocarcinoma	5.0 x 1.0	II	No	No	MSS	No
CRC stage II patient 5	Female	Adenocarcinoma	2.0 x 1.0	II	No	No	MSS	No
CRC stage II patient 6	Male	Adenocarcinoma	4.0 x 1.8	II	No	No	MSS	No
CRC stage II patient 7	Male	Adenocarcinoma	5.5 x 1.0	II	No	No	MSS	No
CRC stage II patient 8	Female	Adenocarcinoma	4.0 x 1.3	II	No	No	MSS	No
CRC stage II patient 9	Male	Adenocarcinoma	4.1 x 1.7	II	No	No	MSS	No
CRC stage II patient 10	Female	Adenocarcinoma	5.2 x 1.3	II	No	No	MSS	No

Abbreviation: AJCC, American Joint Committee on Cancer; LN, lymph node; MSI, microsatellite instability.

Table S3 Clinical characteristics of 13 healthy people enrolled in our study.

Patient No.	Sex
Healthy person 1	Male
Healthy person 2	Male
Healthy person 3	Male
Healthy person 4	Male
Healthy person 5	Male
Healthy person 6	Male
Healthy person 7	Male
Healthy person 8	Male
Healthy person 9	Female
Healthy person 10	Female
Healthy person 11	Male
Healthy person 12	Male
Healthy person 13	Male

gene.name	uniprot.id	ENTREZID	protein name	Alias
EDIL3	O43854	10085	EGF like repeats and discoidin d	DEL1/EDIL3
CD151	P48509	977	CD151 molecule (Raph blood gr	CD151/GP27/MER2/PETA-3/RAPH/SFA1/TSPAN24
RETN	Q9HD89	56729	resistin	ADSF/FIZZ3/RETN/RETN1/RSTN/XCP1
ITGA1	P56199	3672	integrin subunit alpha 1	CD49a/ITGA1/VLA1
ITGA8	P53708	8516	integrin subunit alpha 8	ITGA8
ITGAX	P20702	3687	integrin subunit alpha X	CD11C/ITGAX/SLEB6
ITGB5	P18084	3693	integrin subunit beta 5	ITGB5
IDH1	O75874	3417	isocitrate dehydrogenase (NADP(	HEL-216/HEL-S-26/IDCD/IDH/IDH1/IDP/IDPC/PICD
CD24	P25063	100133941	CD24 molecule	CD24/CD24A
BSG	P35613	682	basigin (Ok blood group)	5F7/BSG/CD147/EMMPRIN/EMPRIN/OK/TCSF
MUC4	Q99102	4585	mucin 4, cell surface associated	ASGP/HSA276359/MUC-4/MUC4
ITGA3	P26006	3675	integrin subunit alpha 3	CD49C/FRP-2/GAP-B3/GAPB3/ILNEB/ITGA3/MSK18/VC
ITGA11	Q9UKX5	22801	integrin subunit alpha 11	HsT18964/ITGA11
ITGA2B	P08514	3674	integrin subunit alpha 2b	BDPLT16/BDPLT2/CD41/CD41B/GP2B/GPIIb/GT/GTA/
ITGB6	P18564	3694	integrin subunit beta 6	AI1H/ITGB6
CLSTN1	O94985	22883	calsyntenin 1	ALC-ALPHA/alcalpha1/alcalpha2/CDHR12/CLSTN1/CST-
KRT18	P05783	3875	keratin 18	CK-18/CYK18/K18/KRT18
RARRES3	Q9UL19	5920	retinoic acid receptor responder	HRASLS4/HRSL4/PLA1/2-3/RARRES3/RIG1/TIG3
ITGA5	P08648	3678	integrin subunit alpha 5	CD49e/FNRA/ITGA5/VLA-5/VLA5A
ITGAE	P38570	3682	integrin subunit alpha E	CD103/HUMINAE/ITGAE
ITGB2	P05107	3689	integrin subunit beta 2	CD18/ITGB2/LAD/LCAMB/LFA-1/MAC-1/MF17/MFI7
ITGB7	P26010	3695	integrin subunit beta 7	ITGB7
ADAM10	O14672	102	ADAM metallopeptidase domain	AD10/AD18/ADAM10/CD156c/CDw156/HsT18717/kuz/
FOLH1	Q04609	2346	folate hydrolase 1	FGCP/FOLH/FOLH1/GCP2/GCPII/mGCP/NAALAD1/NAA
EPHA2	P29317	1969	EPH receptor A2	ARCC2/CTPA/CTPP1/CTRCT6/ECK/EPHA2
ITGA6	P23229	3655	integrin subunit alpha 6	CD49f/ITGA6/ITGA6B/VLA-6
ITGAL	P20701	3683	integrin subunit alpha L	CD11A/ITGAL/LFA-1/LFA1A
CDCP1	Q9H5V8	64866	CUB domain containing protein	CD318/CDCP1/SIMA135/TRASK
ITGB8	P26012	3696	integrin subunit beta 8	ITGB8
PLAU	P00749	5328	plasminogen activator, urokinase	ATF/BDPLT5/PLAU/QPD/u-PA/UPA/URK
ITGB3	P05106	3690	integrin subunit beta 3	BDPLT16/BDPLT2/CD61/GP3A/GPIIIa/GT/ITGB3
TPBG	Q13641	7162	trophoblast glycoprotein	5T4/5T4AG/M6P1/TPBG/WAIF1
ITGA2	P17301	3673	integrin subunit alpha 2	BR/CD49B/GPIa/HPA-5/ITGA2/VLA-2/VLAA2
ITGA7	Q13683	3679	integrin subunit alpha 7	ITGA7
ITGAV	P06756	3685	integrin subunit alpha V	CD51/ITGAV/MSK8/VNRA/VTNR
ITGB4	P16144	3691	integrin subunit beta 4	CD104/GP150/ITGB4
EGF	P01133	1950	epidermal growth factor	EGF/HOMG4/URG
CD163	Q86VB7	9332	CD163 molecule	CD163/M130/MM130/SCARI1
MET	P08581	4233	MET proto-oncogene, receptor t	AUTS9/c-Met/DFNB97/HGFR/MET/RCCP2
L1CAM	P32004	3897	L1 cell adhesion molecule	CAML1/CD171/HSAS/HSAS1/L1CAM/MASA/MIC5/N-CAM
NT5E	P21589	4907	5'-nucleotidase ecto	CALJA/CD73/E5NT/eN/eNT/NT/NT5/NT5E/NTE
PDGFRA	P16234	5156	platelet derived growth factor rec	CD140A/PDGFR-2/PDGFR2/PDGFRA
TIMP1	P01033	7076	TIMP metallopeptidase inhibitor	CLGI/EPA/EPO/HCI/TIMP/TIMP-1/TIMP1
TGFB1	P01137	7040	transforming growth factor beta	CED/DPD1/LAP/TGFB/TGFB1/TGFbeta
VEGFA	P15692	7422	vascular endothelial growth facto	MVCD1/VEGF/VEGFA/VPF
ANG	P03950	283	angiogenin	ALS9/ANG/HEL168/RAA1/RNASE4/RNASE5
CEACAM1	P13688	634	carcinoembryonic antigen relatec	BGP/BGP1/BGPI/CEACAM1
CD63	P08962	967	CD63 molecule	CD63/LAMP-3/ME491/MLA1/OMA81H/TSPAN30
PDPN	Q86YL7	10630	podoplanin	AGGRUS/GP36/Gp38/GP40/HT1A-1/OTS8/PA2.26/PDP
ANXA1	P04083	301	annexin A1	ANX1/ANXA1/LPC1
ALCAM	Q13740	214	activated leukocyte cell adhesion	ALCAM/CD166/MEMD
CXCR4	P61073	7852	C-X-C motif chemokine receptor	CD184/CXCR4/D2S201E/FB22/HM89/HSY3RR/LAP-3/L
GDF15	Q99988	9518	growth differentiation factor 15	GDF-15/GDF15/MIC-1/MIC1/NAG-1/PDF/PLAB/PTGFB
CD9	P21926	928	CD9 molecule	BTCC-1/CD9/DRAP-27/MIC3/MRP-1/TSPAN-29/TSPAN2
EGFR	P00533	1956	epidermal growth factor receptor	EGFR/ERBB/ERBB1/HER1/mENA/NISBD2/PIG61
ITGB1	P05556	3688	integrin subunit beta 1	CD29/FNRB/GPIIA/ITGB1/MDF2/MSK12/VLA-BETA/VLA
HSP90AA1	P07900	3320	heat shock protein 90 alpha fam	EL52/HEL-S-65p/Hsp103/HSP86/Hsp89/HSP89A/Hsp90
MMP14	P50281	4323	matrix metallopeptidase 14	MMP-14/MMP-X1/MMP14/MT-MMP/MT-MMP 1/MT1-M
THY1	P04216	7070	Thy-1 cell surface antigen	CD90/CDw90/THY1
ABCG2	Q9UNQ0	9429	ATP binding cassette subfamily (	ABC15/ABCG2/ABCP/BCRP/BCRP1/BMDP/CD338/CDw
MMP9	P14780	4318	matrix metallopeptidase 9	CLG4B/GELB/MANDP2/MMP-9/MMP9
EPCAM	P16422	4072	epithelial cell adhesion molecule	DIAR5/EGP-2/EGP314/EGP40/EPCAM/ESA/HNPCC8/KS
CXCL16	Q9H2A7	58191	C-X-C motif chemokine ligand 1	CXCL16/CXCLG16/SR-PSOX/SRPSOX
ITGAM	P11215	3684	integrin subunit alpha M	CD11B/CR3A/ITGAM/MAC-1/MAC1A/MO1A/SLEB6
HSPA1A	P0DMV8	3303	heat shock protein family A (Hsp	HEL-S-103/HSP70-1/HSP70-1A/HSP70.1/HSP70I/HSP7
ENG	P17813	2022	endoglin	END/ENG/HHT1/ORW1
CDH2	P19022	1000	cadherin 2	CD325/CDH2/CDHN/CDw325/NCAD
NES	P48681	10763	nestin	Nbla00170/NES
MMP2	P08253	4313	matrix metallopeptidase 2	CLG4/CLG4A/MMP-2/MMP-II/MMP2/MONA/TBE-1
CD36	P16671	948	CD36 molecule	BDPLT10/CD36/CHDS7/FAT/GP3B/GP4/GPIV/PASIV/SC
ITGA9	Q13797	3680	integrin subunit alpha 9	ALPHA-RLC/ITGA4L/ITGA9/RLC
CAV1	Q03135	857	caveolin 1	BSCL3/CAV1/CGL3/LCCNS/MSTP085/PPH3/VIP21
CCR6	P51684	1235	C-C motif chemokine receptor 6	BN-1/C-C CKR-6/CC-CKR-6/CCR-6/CCR6/CD196/CKR-
MUC1	P15941	4582	mucin 1, cell surface associated	ADMCKD/ADMCKD1/CA 15-3/CD227/EMA/H23AG/KL-6
LGR5	O75473	8549	leucine rich repeat containing G	FEX/GPR49/GPR67/GRP49/HG38/LGR5
ALDH1A1	P00352	216	aldehyde dehydrogenase 1 famil	ALDC/ALDH-E1/ALDH1/ALDH11/ALDH1A1/HEL-9/HEL-S
CXCL8	P10145	3576	C-X-C motif chemokine ligand 8	CXCL8/GCP-1/GCP1/IL8/LECT/LUCT/LYNAP/MDNCF/M
EFNB2	P52799	1948	ephrin B2	EFNB2/EPLG5/Htk-L/HTKL/LERK5
GPC1	P35052	2817	glypican 1	glypican/GPC1
MCAM	P43121	4162	melanoma cell adhesion molecu	CD146/MCAM/MUC18
ERBB2	P04626	2064	erb-b2 receptor tyrosine kinase 2	CD340/ERBB2/HER-2/HER-2/neu/HER2/MLN 19/NEU/N
LAMP1	P11279	3916	lysosomal associated membrane	CD107a/LAMP1/LAMPA/LGP120
TACSTD2	P09758	4070	tumor associated calcium signal	EGP-1/EGP1/GA733-1/GA7331/GP50/M1S1/TACSTD2/
ANPEP	P15144	290	alanyl aminopeptidase, membrar	ANPEP/APN/CD13/GP150/LAP1/P150/PEPN
KIT	P10721	3815	KIT proto-oncogene receptor tyr	C-Kit/CD117/KIT/PBT/SCFR
FGF2	P09038	2247	fibroblast growth factor 2	BFGF/FGF-2/FGF2/FGFB/HBGF-2
TIMP2	P16035	7077	TIMP metallopeptidase inhibitor	CSC-21K/DDC8/TIMP2
IL6	P05231	3569	interleukin 6	BSF-2/BSF2/CDF/HGF/HSF/IFN-beta-2/IFNB2/IL-6/IL6
FN1	P02751	2335	fibronectin 1	CIG/ED-B/FINC/FN/FN1/FNZ/GFND/GFND2/LETS/MSF/
CCR2	P41597	729230	C-C motif chemokine receptor 2	CC-CKR-2/CCR-2/CCR2/CCR2A/CCR2B/CD192/CKR2/
XIAP	P98170	331	X-linked inhibitor of apoptosis	API3/BIRC4/hIAP-3/hIAP3/IAP-3/ILP1/MIHA/XIAP/XLP2
CDH17	Q12864	1015	cadherin 17	CDH16/CDH17/HPT-1/HPT1
DPP4	P27487	1803	dipeptidyl peptidase 4	ADABP/ADCP2/CD26/DPP4/DPPIV/TP103
TRAF3	Q13114	7187	TNF receptor associated factor 3	CAP-1/CAP1/CD40bp/CRAF1/IIAE5/LAP1/RNF118/TRAF
CLDN1	O95832	9076	claudin 1	CLD1/CLDN1/ILVASC/SEMP1
LAMP2	P13473	3920	lysosomal associated membrane	CD107b/LAMP-2/LAMP2/LAMPB/LGP-96/LGP110
HLA-DRA	P01903	3122	major histocompatibility complex	HLA-DRA/HLA-DRA1
ANXA2	P07355	302	annexin A2	ANX2/ANX2L4/ANXA2/CAL1H/HEL-S-270/LIP2/LPC2/LP
PROM1	O43490	8842	prominin 1	AC133/CD133/CORD12/MCDR2/MSTP061/PROM1/PRO
TSPAN8	P19075	7103	tetraspanin 8	CO-029/TM4SF3/TSPAN8
ICAM1	P05362	3383	intercellular adhesion molecule 1	BB2/CD54/ICAM1/P3.58
NGFR	P08138	4804	nerve growth factor receptor	CD271/Gp80-LNGFR/NGFR/p75(NTR)/p75NTR/TNFRSF
MS4A1	P11836	931	membrane spanning 4-domains	B1/Bp35/CD20/CVID5/LEU-16/MS4A1/MS4A2/S7
CD44	P16070	960	CD44 molecule (Indian blood gr	CD44/CDW44/CSPG8/ECMR-III/HCELL/HUTCH-I/IN/LHF
ABCB5	Q2M3G0	340273	ATP binding cassette subfamily I	ABCB5/ABCB5alpha/ABCB5beta/EST422562
ILK	Q13418	3611	integrin linked kinase	HEL-S-28/ILK/ILK-1/ILK-2/P59/p59ILK
BIRC5	O15392	332	baculoviral IAP repeat containing	API4/BIRC5/EPR-1
WNT11	O96014	7481	Wnt family member 11	HWNT11/WNT11
PTK2	Q05397	5747	protein tyrosine kinase 2	FADK/FAK/FAK1/FRNK/p125FAK/pp125FAK/PPP1R71/
FASN	P49327	2194	fatty acid synthase	FAS/FASN/OA-519/SDR27X1
PKM	P14618	5315	pyruvate kinase M1/2	CTHBP/HEL-S-30/OIP3/PK3/PKM/PKM2/TCB/THBP1
RIOX2	Q8IUF8	84864	ribosomal oxygenase 2	JMJD10/MDIG/MINA/MINA53/NO52/RIOX2/ROX
MAP2K4	P45985	6416	mitogen-activated protein kinase	JNKK/JNKK1/MAP2K4/MAPKK4/MEK4/MKK4/PRKMK4
EPS8	Q12929	2059	epidermal growth factor receptor	DFNB102/EPS8
ALPP	P05187	250	alkaline phosphatase, placental	ALP/ALPP/PALP/PLAP/PLAP-1

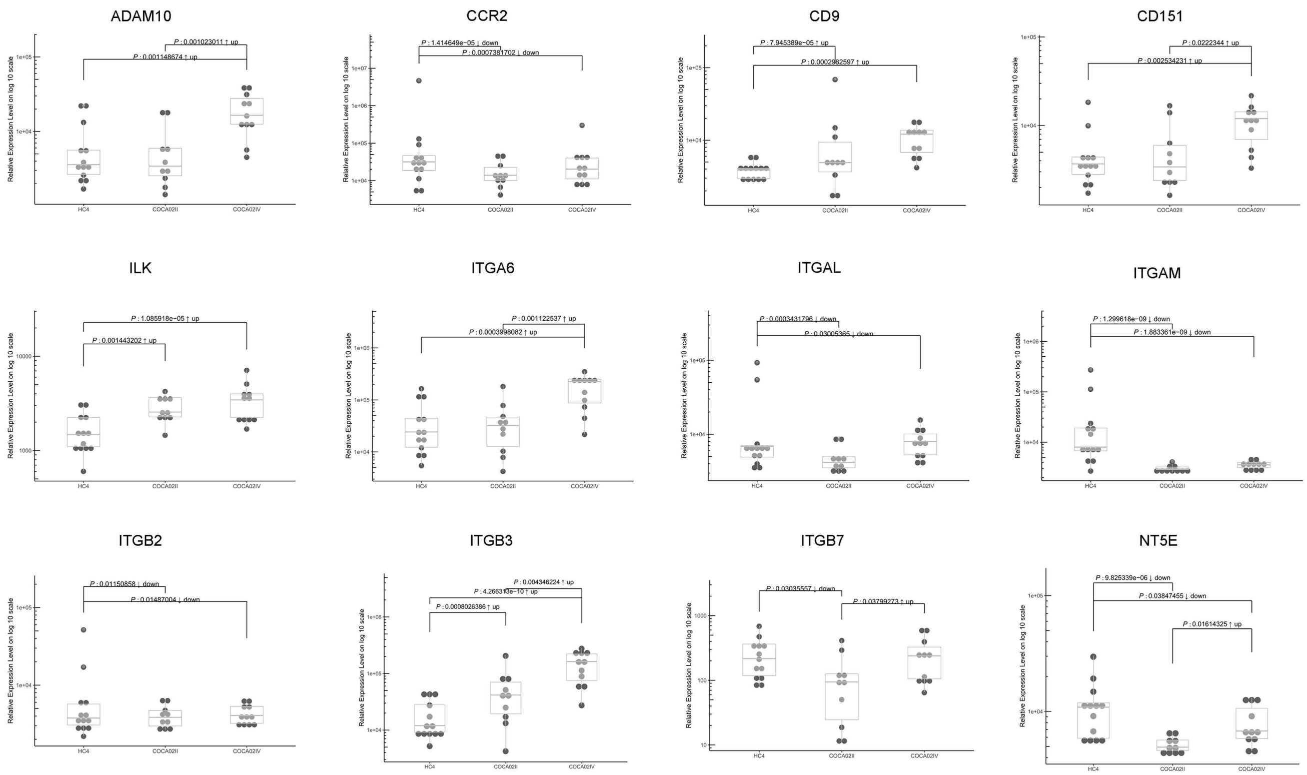

ADAM10
P : 0.001023011 ↑ up
P : 0.001148674 ↑ up
CCR2
P : 1.414649e-05 ↓ down
P : 0.0007381702 ↓ down
CD9
P : 7.945389e-05 ↑ up
CD151
P : 0.0222344 ↑ up
P : 0.002534231 ↑ up
ILK
P : 1.085918e-05 ↑ up
P : 0.001443202 ↑ up
ITGA6
P : 0.001122537 ↑ up
P : 0.0003998082 ↑ up
ITGAL
ITGAM
P : 1.299618e-09 ↓ down
P : 1.883361e-09 ↓ down
ITGB2
P : 0.01150858 ↓ down
P : 0.01487004 ↓ down
ITGB3
P : 0.004346224 ↑ up
P : 0.0008026386 ↑ up
ITGB7
NT5E
P : 9.825339e-06 ↓ down
P : 0.03847455 ↓ down
P : 0.01614325 ↑ up
Relative Expression Level on log 10 scale
HC4
COCA02II
COCA02IV

Figure S5

S6 differential expression of proteins

	log2FC	log2C	PValue	FDR	Style
**ITGAM**	-3.672960381	14.06734535	1.30E-09	1.48E-07	down
**NT5E**	-1.125087871	13.05862415	9.83E-06	0.000537566	down
**CCR2**	-4.383675942	17.43783243	1.41E-05	0.000537566	down
**CD9**	1.661482559	13.07914949	7.95E-05	0.002093669	up
**THY1**	-0.799085359	12.58406788	9.18E-05	0.002093669	down
**ITGAL**	-1.746537343	13.34073654	0.00034318	0.006520413	down
**EPS8**	0.61790542	11.3544918	0.000771656	0.0114376	up
**ITGB3**	1.551220903	16.16572096	0.000802639	0.0114376	up
**ILK**	0.794359073	11.34326061	0.001443202	0.018280557	up
**TIMP1**	-0.707274976	12.98341191	0.002220921	0.023910598	down
**MMP9**	-0.559448384	12.32739094	0.002307163	0.023910598	down
**RIOX2**	0.561759534	10.89587334	0.004281381	0.040673117	up

	log2FC	log2C	PValue	FDR	Style
**ITGB3**	2.958128958	16.16572096	4.27E-10	4.86E-08	up
**ITGAM**	-3.455534956	14.06734535	1.88E-09	1.07E-07	down
**ILK**	1.075877152	11.34326061	1.09E-05	0.000412649	up
**BIRC5**	0.731579421	11.02716115	6.94E-05	0.001978133	up
**CD9**	1.49135119	13.07914949	0.00029826	0.006800322	up
**ITGA6**	1.924163655	16.45098368	0.000399808	0.007596356	up
**CCR2**	-3.059789801	17.43783243	0.00073817	0.012021628	down
**ADAM10**	1.509053321	13.44801144	0.001148674	0.016328447	up
**EPS8**	0.578623354	11.3544918	0.001289088	0.016328447	up
**CD151**	1.178513061	12.83020882	0.002534231	0.026685795	up
**TIMP1**	-0.675937292	12.98341191	0.002574945	0.026685795	down
**CEACAM1**	-0.397561717	11.73733433	0.003214383	0.030536636	down
**CD163**	-0.322316357	11.89133898	0.004103146	0.035981431	down
**HSPA1A**	-0.450704378	12.04384634	0.005313006	0.043263048	down
**THY1**	-0.52531521	12.58406788	0.00766247	0.058234769	down

	log2FC	log2C	PValue	FDR	Style
**EGF**	-0.417688795	11.55286841	0.000153086	0.017451785	down
**ADAM10**	1.670041567	13.44801144	0.001023011	0.042656424	up
**ITGA6**	1.940860659	16.45098368	0.001122537	0.042656424	up

Supplementary meterials S7 Cluster annotation of plasma exosomes

Cluster type	Marker1	Marker2	Marker3	Annotation
Cluster1(6%)	CCR2			Myeloid-derived
Cluster2(5.49%)	ERBB2			Cancer cells-derived
Cluster3(8.41%)	NO			Unknown
Cluster4(32.85%)	ITGB3	ITGA6		Cancer cells-derived
Cluster5(10.72%)	CDH17	MCAM	MUC1	Cancer cells-derived
Cluster6(4.96%)	FN1			Mesenchymal cells-derived
Cluster7(13.09%)	NO			Unknown
Cluster8(3.6%)	ITGB3			Cancer cells-derived
Cluter9(3.51%)	RETN			Mature fat cells-derived
Cluster10(3.78%)	XIAP			Cancer cells-derived
Cluster11(4.58%)	ITGAM	ITGAL	ITGB2	Monocyte/macrophage-derived
Cluster12(3.01%)	ITGA6			Cancer cells-derived

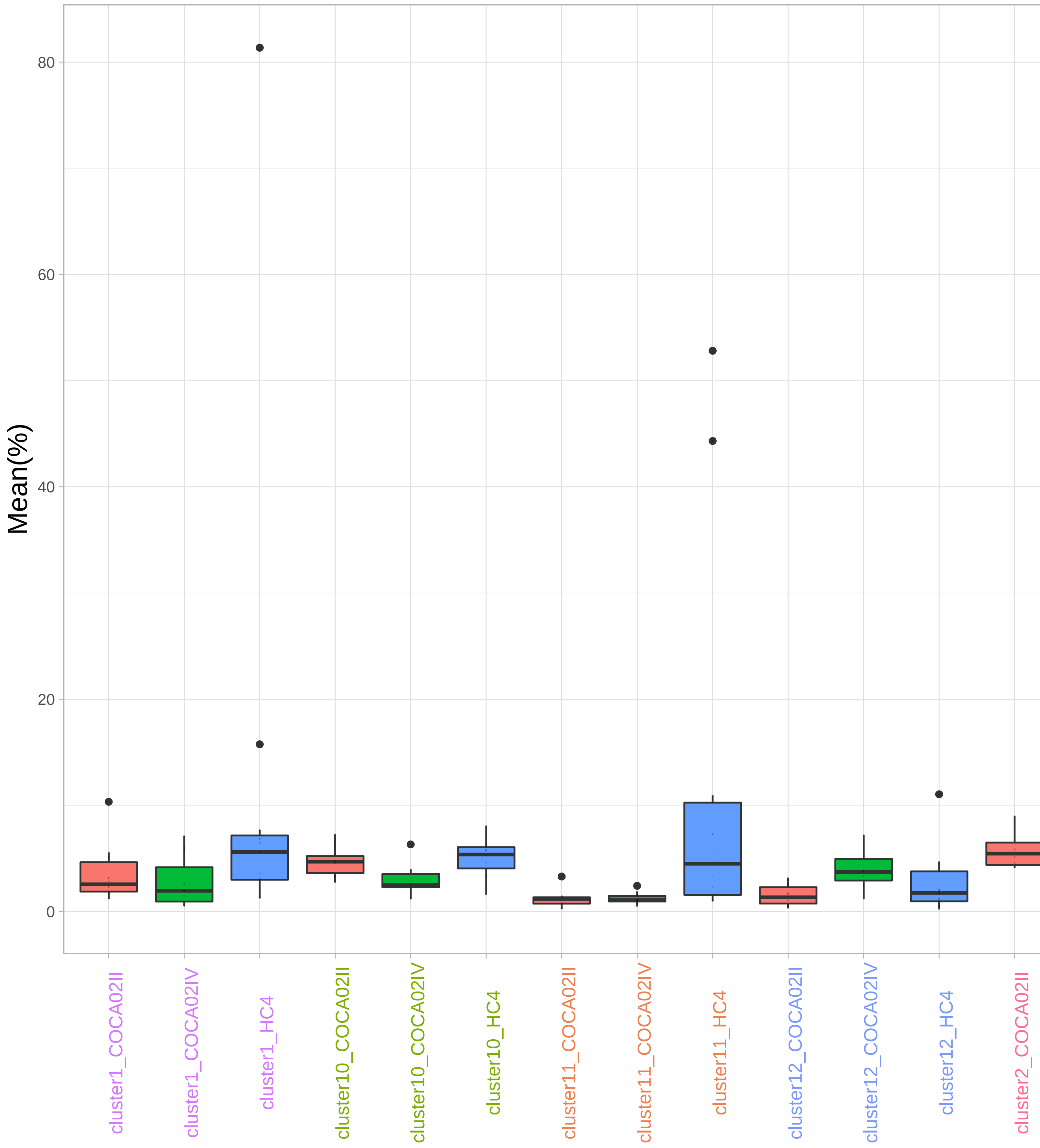
Mean(%)
80
60
40
20
0
cluster1_COCA02II
cluster1_COCA02IV
cluster1_HC4
cluster10_COCA02II
cluster10_COCA02IV
cluster10_HC4
cluster11_COCA02II
cluster11_COCA02IV
cluster11_HC4
cluster12_COCA02II
cluster12_COCA02IV
cluster12_HC4
cluster2_COCA02II

Table S9 Clinical characteristics from 6 patients with premalignant lesions enrolled in our study.

Patient No.	Sex	Age	Pathology
patient 1	Male	49	Low grade tubular adenoma
patient 2	Male	82	Low grade tubular adenoma
patient 3	Male	58	Low grade tubular adenoma
patient 4	Female	69	Low grade tubular adenoma
patient 5	Female	56	Low grade tubular adenoma
patient 6	Female	64	Low grade tubular adenoma

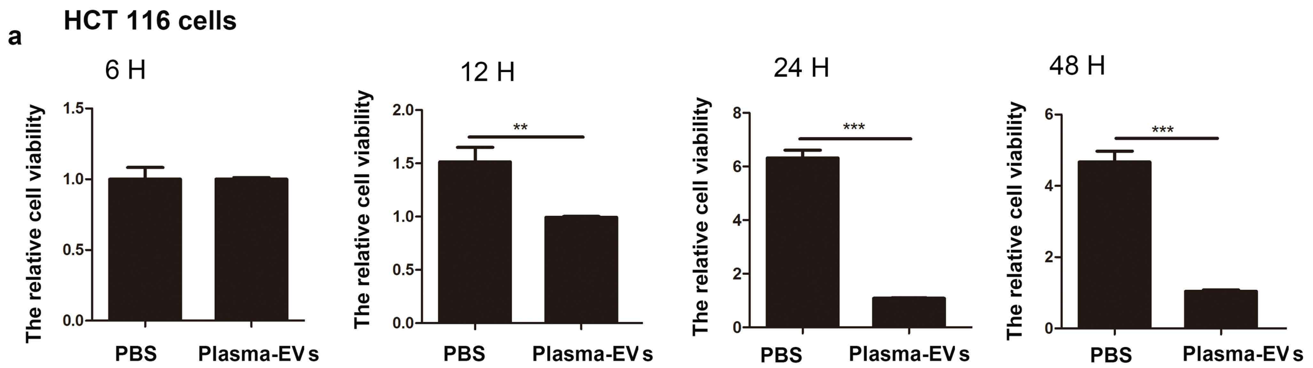
a
HCT 116 cells
6 H
12 H
24 H
48 H
The relative cell viability
1.5
1.0
0.5
0.0
2.0
8
6
4
2
0
**
***
***
PBS
Plasma-EVs

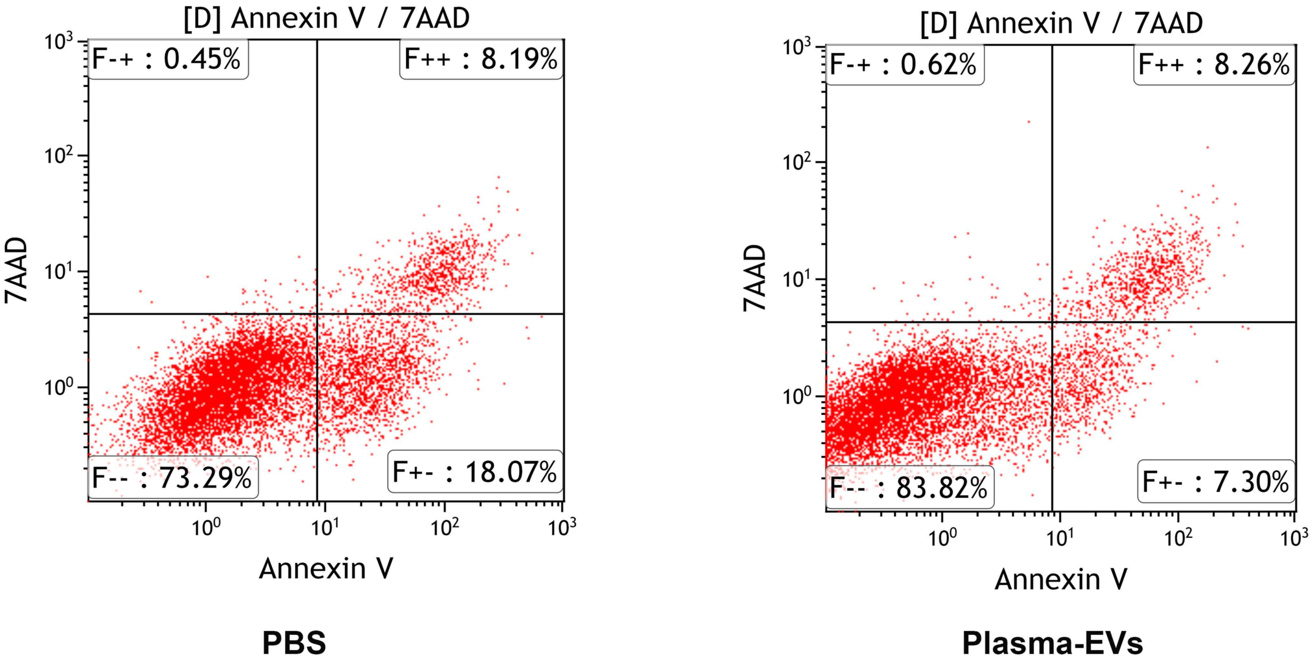
[D] Annexin V / 7AAD
F-+ : 0.45%
F++ : 8.19%
F-- : 73.29%
F+- : 18.07%
7AAD
Annexin V
PBS
[D] Annexin V / 7AAD
F-+ : 0.62%
F++ : 8.26%
F-- : 83.82%
F+- : 7.30%
7AAD
Annexin V
Plasma-EVs

S12 validation cohort: exosome subpopulations and their proteomic profiles

1. Contribution_Rate_of_Proteins_in_Each_Cluster

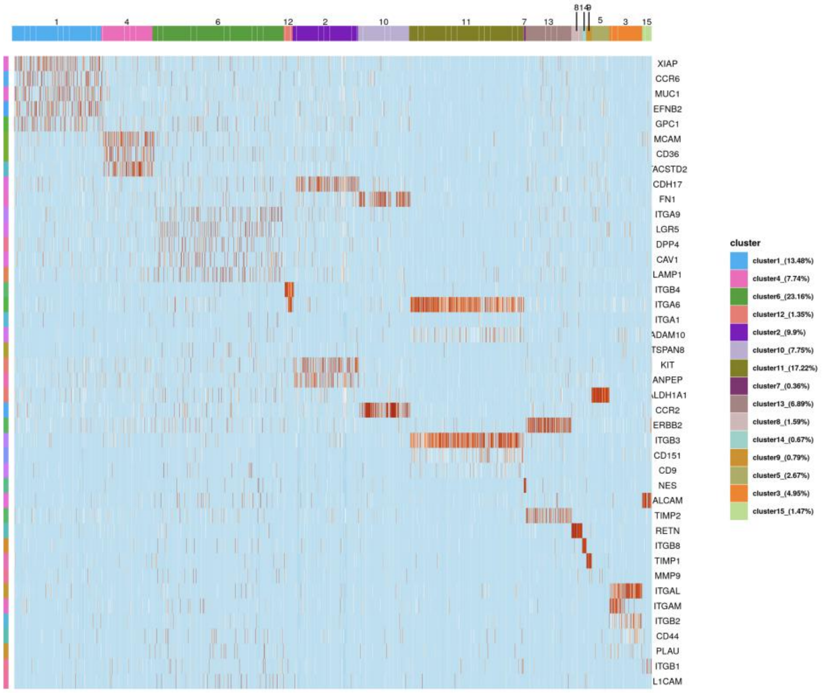

2. Tsne_Plot_of_Each_Groups_flowsom

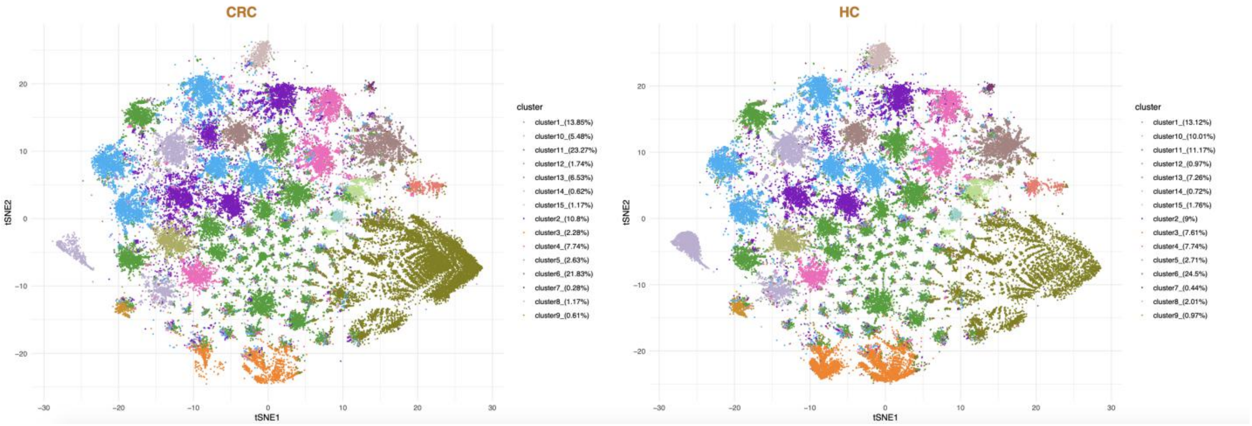

3. Proportion of cluster 3 and 11 in total detected exosomes

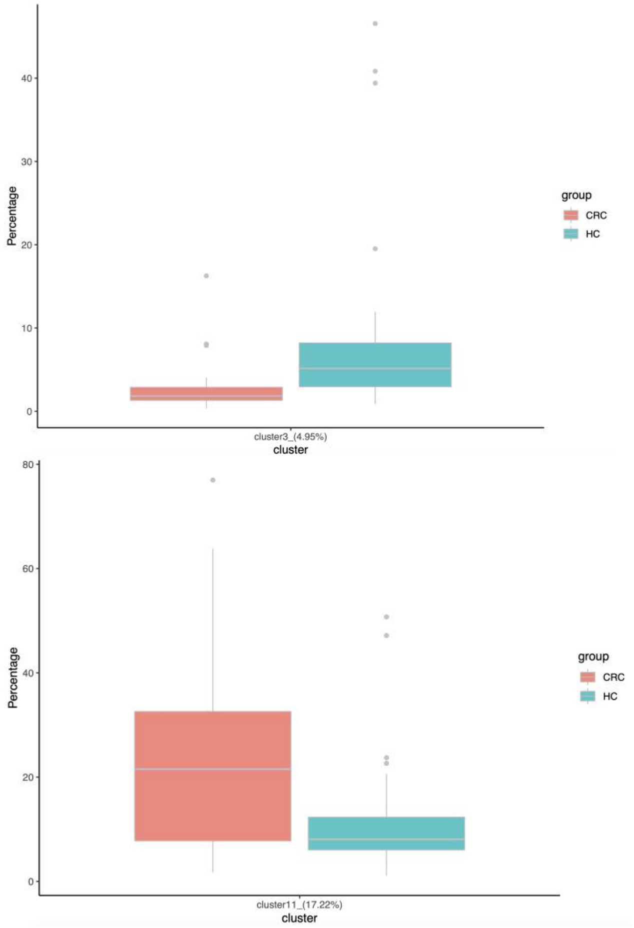

4. Proteomic profile of cluster 3

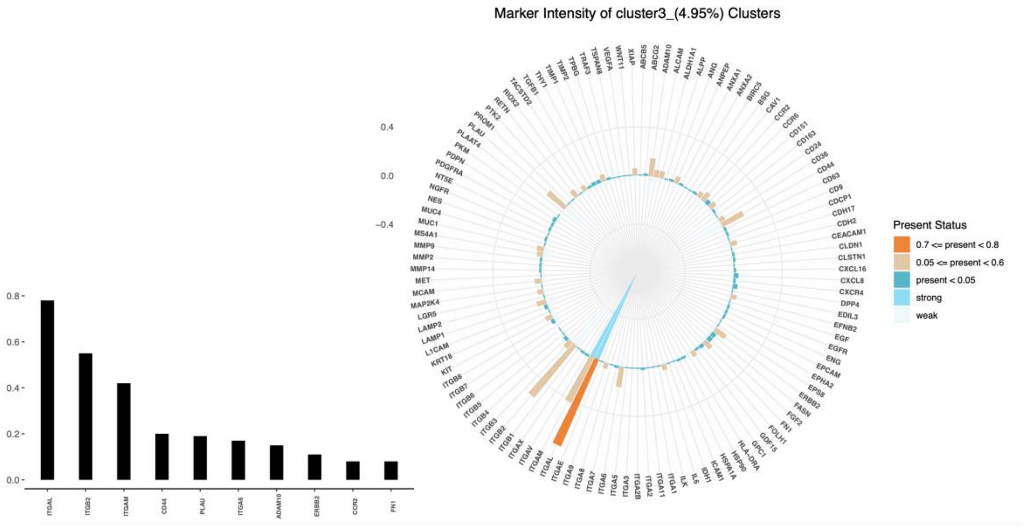

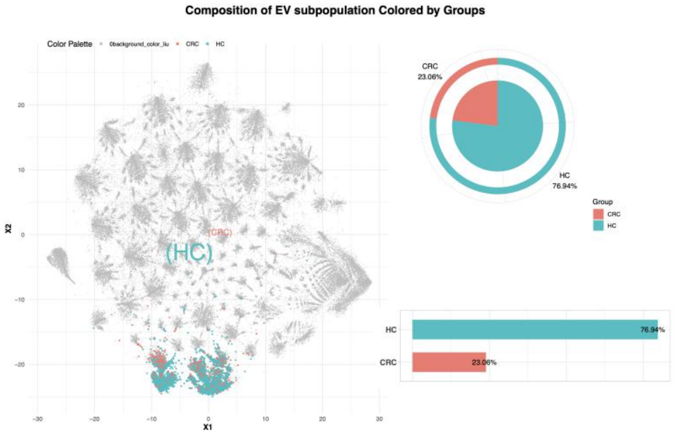

5. Proteomic profile of cluster 11

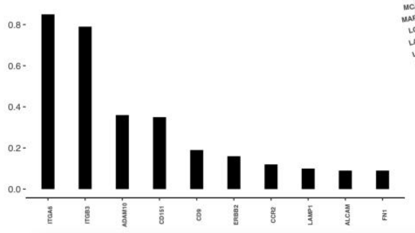

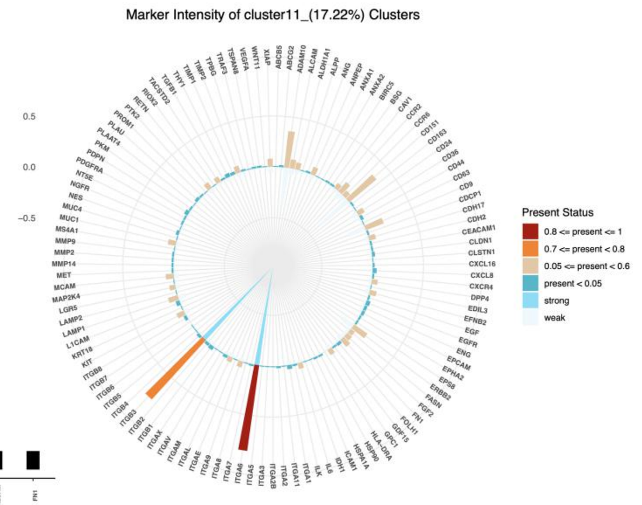

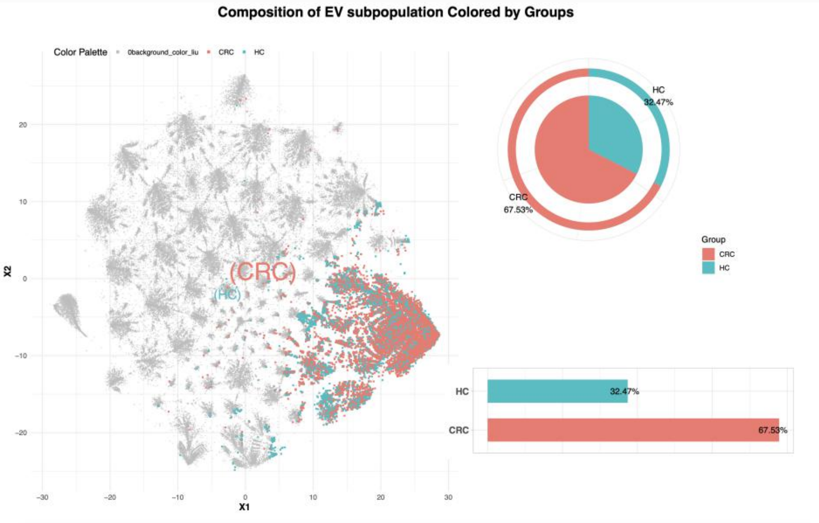

Supplementary materials S13 information of validation cohort

patient	gender	age	location	pathology	AJCC stage	metastasis to lymph node	distant metastasis
patient1	F	55	Transverse colon	adenoma	IIA	No	No
patient2	F	68	Transverse colon	adenoma	II	No	No
patient3	F	70	Transverse colon	adenoma	IIC	No	No
patient4	M	57	descending colon	adenoma	IIA	No	No
patient5	F	64	colon	adenoma	III	Yes	No
patient6	F	81	Transverse colon	adenoma	I	No	No
patient7	M	39	ascending colon	adenoma	I	No	No
patient8	M	48	sigmoid colon	adenoma	IIIB	Yes	No
patient9	F	82	sigmoid colon	adenoma	IIA	No	No
patient10	F	65	sigmoid colon	adenoma	IIA	No	No
patient11	M	48	sigmoid colon	adenoma	IIA	No	No
patient12	F	36	sigmoid colon	adenoma	IIIB	Yes	No
patient13	F	75	sigmoid colon	adenoma	IIIB	Yes	No
patient14	M	70	sigmoid colon	adenoma	I	No	No
patient15	M	45	Right-sided colon	adenoma	II	No	No
patient16	F	64	Right-sided colon	adenoma	I	No	No
patient17	M	52	Right-sided colon	adenoma	IIIB	Yes	No
patient18	M	47	rectum	adenoma	IIIB	Yes	No
patient19	M	56	rectum	adenoma	II	No	No
patient20	M	70	rectum	adenoma	IIIB	Yes	No
patient21	F	64	rectum	adenoma	I	No	No
patient22	M	39	rectum	adenoma	II	No	No
patient23	M	56	rectum	adenoma	I	No	No
patient24	F	85	rectum	adenoma	IV	Yes	Yes
patient25	F	64	sigmoid colon	adenoma	IIA	No	No
patient26	F	69	rectum	adenoma	IIIA	Yes	No
patient27	M	52	rectum	adenoma	I	No	No
patient28	M	34	rectum	adenoma	III	Yes	No
patient29	F	60	rectum	adenoma	IIIB	Yes	No
patient30	M	45	rectum	adenoma	IIB	No	No

Healthy donor	age	gender	Healthy donor	age	gender
HC1	23	F	HC26	23	M
HC2	24	F	HC27	29	M
HC3	26	F	HC28	30	M
HC4	26	F	HC29	31	M
HC5	27	F	HC30	31	M
HC6	27	F	HC31	31	M
HC7	28	F	HC32	32	M
HC8	29	F	HC33	34	M
HC9	29	F	HC34	36	M
HC10	33	F	HC35	38	M
HC11	34	F	HC36	32	M
HC12	35	F	HC37	39	M
HC13	36	F	HC38	42	M
HC14	38	F	HC39	43	M
HC15	39	F	HC40	46	M
HC16	39	F	HC41	47	M
HC17	39	F	HC42	48	M
HC18	42	F	HC43	49	M
HC19	43	F	HC44	50	M
HC20	43	F	HC45	53	M
HC21	45	F	HC46	67	M
HC22	47	F	HC47	78	M
HC23	50	F	HC48	83	M
HC24	52	F	HC49	28	F
HC25	48	F	HC50	33	M

S14

protein name	gene name	Synonym
ATP binding cassette subfamily G member 2	ABCG2	ABCP, BCRP, CD338, EST157481, MXR
Disintegrin and metalloproteinase domain-containing protein 10	ADAM10	CD156C, HsT18717, kuz, MADM
adhesion G protein-coupled receptor G1	ADGRG1	GPR56, TM7LN4, TM7XN1
adiponectin	ADIPOQ	ACDC, ACRP30, adiponectin, apM1, GBP28
activated leukocyte cell adhesion molecule	ALCAM	CD166, MEMD
Retinal dehydrogenase 1	ALDH1A1	ALDH1, PUMB1, RALDH1
Amphoterin-induced protein 1	AMIGO1	AMIGO, KIAA1163
Amphoterin-induced protein 2	AMIGO2	ALI1, DEGA
alanyl aminopeptidase, membrane	ANPEP	CD13, gp150, LAP1, p150, PEPN
annexin A1	ANXA1	ANX1, LPC1
AXL receptor tyrosine kinase receptor UFO	AXL	ARK, JTK11, Tyro7, UFO
basal cell adhesion molecule	BCAM	CD239, LU
BOC cell adhesion associated, oncogene regulated	BOC	CDON2
Cell adhesion molecule 3	CADM3	BIgR, FLJ10698, IGSF4B, Necl-1, NECL1, SynCAM3, TSLL1
cell adhesion molecule 4	CADM4	IGSF4C, Necl-4, SynCAM4, TSLL2
Caveolin-1	CAV1	CAV
C-C motif chemokine receptor 2	CCR2	CC-CKR-2, CD192, CKR2, CMKBR2, FLJ78302, MCP-1-[illegible]
C-C motif chemokine receptor 6	CCR6	BN-1, CD196, CKR-L3, CMKBR6, DCR2, DRY-6, GPR-CY4, GPR29, STRL22
CD151 antigen	CD151	PETA-3, RAPH, SFA-1, TSPAN24
CD20	CD20	B1, Bp35, CD20, MS4A2
CD22 molecule	CD22	SIGLEC-2, SIGLEC2
Signal transducer CD24	CD24	CD24A
CD26/Dipeptidyl peptidase 4	CD26	ADCP2, CD26, DPPIV
CD27 molecule	CD27	S152, TNFRSF7, Tp55
CD274 molecule	CD274	B7-H, B7-H1, B7H1, PD-L1, PDCD1LG1, PDL1
CD33 molecule	CD33	FLJ00391, p67, SIGLEC-3, SIGLEC3
CD36/Platelet glycoprotein 4	CD36	FAT, GP3B, GP4, GPIV, SCARB3
CD44 antigen	CD44	CD44R, CSPG8, HCELL, IN, MC56, MDU2, MDU3, MIC4, Pgp1
CD63 antigen	CD63	ME491, MLA1, TSPAN30
CD81 antigen	CD81	TAPA-1, TAPA1, TSPAN28
CD9 antigen	CD9	BA2, MIC3, MRP-1, P24, TSPAN29
CUB domain-containing protein 1	CDCP1	CD318, SIMA135
cadherin 1	CDH1	CD324, UVO, uvomorulin
cadherin 11	CDH11	CAD11, OB
cadherin 12	CDH12	Br-cadherin, CDHB
cadherin 13	CDH13	CDHH
Cadherin-15	CDH15	CDH14, CDH3
Cadherin-17	CDH17	cadherin, HPT-1
Cadherin-2	CDH2	CD325, CDHN, NCAD
cadherin 3	CDH3	CDHP, PCAD
cadherin 4	CDH4	
cadherin 5	CDH5	7B4, CD144
cadherin 6	CDH6	
Cadherin-related family member 5	CDHR5	FLJ20219, MU-PCDH, MUCDHL, MUPCDH
Cell adhesion molecule-related/down-regulated by oncogenes	CDON	CDO, CDON1, ORCAM
CEA cell adhesion molecule 1	CEACAM1	BGP, BGP1, CD66a
CEA cell adhesion molecule 3	CEACAM3	CD66d, CGM1
Carcinoembryonic antigen-related cell adhesion molecule 4	CEACAM4	CGM7
CEA cell adhesion molecule 5	CEACAM5	CD66e, CEA
Carcinoembryonic antigen-related cell adhesion molecule 6	CEACAM6	CD66c, NCA
CEA cell adhesion molecule 7	CEACAM7	CEA, CGM2
Carcinoembryonic antigen-related cell adhesion molecule 8	CEACAM8	CD66b, CGM6
claudin 1	CLDN1	ILVASC, SEMP1
claudin 10	CLDN10	CPETRL3, OSP-L
claudin 11	CLDN11	OSP, OTM
claudin 12	CLDN12	
claudin 17	CLDN17	MGC126552, MGC126554
Claudin-19	CLDN19	
claudin 3	CLDN3	C7orf1, CPE-R2, CPETR2, HRVP1, RVP1
Claudin-4	CLDN4	CPE-R, CPETR, CPETR1, hCPE-R, WBSCR8
claudin 6	CLDN6	
claudin 8	CLDN8	
C-type lectin domain family 1 member B	CLEC1B	CLEC2
C-type lectin domain family 2 member A	CLEC2A	INPE5792, KACL, PILAR, UNQ5792
C-type lectin domain containing 5A	CLEC5A	CLECSF5, MDL-1
CXADR like membrane protein	CLMP	ACAM, ASAM, FLJ22415
Calsyntenin-1	CLSTN1	CDHR12, CSTN1, KIAA0911
carboxypeptidase M	CPM	
chondroitin sulfate proteoglycan 4	CSPG4	CSPG4A, HMW-MAA, MCSP, MCSPG, MEL-CSPG, MSK16, NG2
Cytotoxic T-lymphocyte protein 4	CTLA4	CD, CD152, CELIAC3, CTLA-4, GSE, IDDM12
CXADR Ig-like cell adhesion molecule	CXADR	CAR
C-X-C motif chemokine ligand 16	CXCL16	CXCLG16, SR-PSOX, SRPSOX
C-X-C motif chemokine ligand 8	CXCL8	3-10C, AMCF-I, b-ENAP, GCP-1, GCP1, IL-8, IL8, K60, LECT, LUCT, LYNAP, MDNCF, MONAP, NAF, NAP-1, NAP1, SCYB8, TSG-1
C-X-C motif chemokine receptor 4	CXCR4	CD184, D2S201E, fusin, HM89, HSY3RR, LESTR, NPY3R, NPYR, NPYY3R
desmocollin 1	DSC1	CDHF1
desmocollin 2	DSC2	CDHF2, DSC3
Desmocollin-3	DSC3	CDHF3, DSC, DSC1, DSC2, DSC4
DS cell adhesion molecule	DSCAM	CHD2-42, CHD2-52
DS cell adhesion molecule like 1	DSCAML1	KIAA1132
Desmoglein-1	DSG1	CDHF4, DSG
Desmoglein-2	DSG2	CDHF5
Desmoglein-3	DSG3	CDHF6
Desmoglein-4	DSG4	CDHF13, LAH
ephrin B2	EFNB2	EPLG5, Htk-L, HTKL, LERK5, MGC126226, MGC126227, MGC126228
Epidermal growth factor receptor	EGFR	ERBB, ERBB1
Endomucin	EMCN	MUC14
Epithelial cell adhesion molecule	EPCAM	17-1A, 323/A3, CD326, CO-17A, EGP-2, EGP34, EGP40, Ep-CAM, ESA, GA733-2, HEA125, KS1/4, KSA, Ly74, M4S1, MH99, MIC18, MK-1, MOC31, TACST-1, TACSTD1, TROP1
EPH receptor A2	EPHA2	ECK
Receptor tyrosine-protein kinase erbB-2	ERBB2	CD340, HER-2, HER2, NEU, NGL
Endothelial cell-selective adhesion molecule	ESAM	W117m
F11 receptor	F11R	CD321, JAM-1, JAM-A, JAM1, JAMA, JCAM, PAM-1
fibronectin 1	FN1	CIG, FINC, GFND2, LETS, MSF
Glutamate carboxypeptidase 2	FOLH1	FOLH, GCP2, GCPII, NAALAD1, NAALAdase, PSM, PSM
Cell surface A33 antigen	GPA33	A33
Glypican-1	GPC1	glypican
gelsolin	GSN	DKFZp313L0718
hepatic and glial cell adhesion molecule	HEPACAM	FLJ25530, GLIALCAM
HLA class I histocompatibility antigen	HLA-A	
HLA class II histocompatibility antigen	HLA-DRA	HLA-DRA1
Intercellular adhesion molecule 1	ICAM1	BB2, CD54
intercellular adhesion molecule 2	ICAM2	CD102
Intercellular adhesion molecule 3	ICAM3	CD50, CDW50, ICAM-R
intercellular adhesion molecule 4	ICAM4	CD242, LW
interleukin 1 receptor accessory protein like 1	IL1RAPL1	IL1R8, IL1RAPL, MRX10, MRX21, MRX34, OPHN4, TIGIRR-2
Interleukin-6	IL6	BSF2, HGF, HSF, IFNB2, IL-6
integrin linked kinase	ILK	
Integrin alpha-1	ITGA1	CD49a, VLA1
Integrin alpha-11	ITGA11	HsT18964
Integrin alpha-2	ITGA2	CD49B
Integrin alpha-2 beta	ITGA2B	CD41, CD41B, GP2B, PPP1R93
Integrin alpha-3	ITGA3	CD49c, GAP-B3, MSK18, VCA-2, VLA3a
Integrin alpha-4	ITGA4	CD49D
Integrin alpha 4 beta 7	ITGA4B7	
Integrin alpha-5	ITGA5	CD49e, FNRA
Integrin alpha-6	ITGA6	CD49f
Integrin alpha-8	ITGA8	
Integrin alpha-9	ITGA9	ALPHA-RLC, ITGA4L, RLC
Integrin alpha-L	ITGAL	CD11A, LFA-1
integrin subunit alpha M	ITGAM	CD11B, CR3A, MAC-1
Integrin alpha-V	ITGAV	CD51, MSK8, VNRA, VTNR
Integrin alpha-X	ITGAX	CD11C
integrin subunit beta 1	ITGB1	CD29, FNRB, GPIIA, MDF2, MSK12
Integrin beta-1	ITGB1	CD29, FNRB, GPIIA, MDF2, MSK12
Integrin beta-2	ITGB2	CD18, LFA-1, MAC-1, MFI7
integrin subunit beta 3	ITGB3	CD61, GP3A, GPIIIa
Integrin beta-4	ITGB4	CD104
Integrin beta-5	ITGB5	
Integrin beta-6	ITGB6	
Integrin beta-7	ITGB7	
Integrin beta-8	ITGB8	
Junctional adhesion molecule B, JAM-B	JAM2	C21orf43, CD322, JAM-B, JAMB, VE-JAM
Junctional adhesion molecule C	JAM3	JAM-C, JAMC
junction adhesion molecule like	JAML	AMICA, AMICA1, Gm638
KIT proto-oncogene, receptor tyrosine kinase	KIT	C-Kit, CD117, PBT, SCFR
Cell Adhesion Molecule L1	L1CAM	CD171, HSAS, HSAS1, MASA, MIC5, S10, SPG1
lymphocyte activating 3	LAG3	CD223
lysosomal associated membrane protein 1	LAMP1	CD107a
Lysosome-associated membrane glycoprotein 2	LAMP2	CD107b
galectin 9	LGALS9	LGALS9A
leucine rich repeat containing G protein-coupled receptor 5	LGR5	FEX, GPR49, GPR67, HG38
Tyrosine-protein kinase Lyn	LYN	JTK8
Mucosal Addressin Cell Adhesion Molecule 1	MADCAM1	MACAM1
melanoma cell adhesion molecule	MCAM	CD146, HEMCAM, MelCAM, METCAM, MUC18
MHC class I polypeptide-related sequence A	MICA	PERB11.1
MHC class I polypeptide-related sequence B	MICB	PERB11.2
matrix metallopeptidase 9	MMP9	CLG4B
mucin 1	MUC1	ADMCKD, ADMCKD1, CD227, MCD, MCKD, MCKD1, PEM, PUM
Mucin-16(Ovarian carcinoma antigen CA125)	MUC16	CA125, FLJ14303
Mucin-19	MUC19	FLJ35746
mucin 4, cell surface associated	MUC4	
neural cell adhesion molecule 1	NCAM1	CD56, NCAM
Nectin-1	NECTIN1	CD111, CLPED1, ED4, HIgR, HVEC, OFC7, PRR, PRR1, PVRL1, PVRR1, SK-12
nectin cell adhesion molecule 2	NECTIN2	CD112, HVEB, PRR2, PVRL2, PVRR2
nectin cell adhesion molecule 3	NECTIN3	CD113, CDw113, DKFZP566B0846, nectin-3, PPR3, PVRL3, PVRR3
nectin cell adhesion molecule 4	NECTIN4	LNIR, nectin-4, PRR4, PVRL4
nestin	NES	FLJ21841
neurofascin	NFASC	FLJ46866, KIAA0756, NF, NRCAML
Tumor necrosis factor receptor superfamily member 16	NGFR	CD271, p75NTR, TNFRSF16
neuroligin 1	NLGN1	KIAA1070
5'-nucleotidase ecto	NT5E	CALJA, CD73, eN, eNT, NT5
neurotrophic receptor tyrosine kinase 3	NTRK3	TRKC
Proteinase-activated receptor 2	PAR2	GPR11, PAR2
protocadherin 1	PCDH1	pc42
Protocadherin-15	PCDH15	CDHR15, DFNB23, USH1F
protocadherin 17	PCDH17	PCDH68, PCH68
protocadherin 19	PCDH19	EFMR, EIEE9, KIAA1313
protocadherin 8	PCDH8	ARCADLIN, PAPC
protocadherin alpha 1	PCDHA1	
protocadherin gamma subfamily C, 3	PCDHGC3	PC-43, PC43, PCDH-GAMMA-C3, PCDH2
programmed cell death 1	PDCD1	CD279, hSLE1, PD-1, PD1, SLEB2
podoplanin	PDPN	aggrus, Gp38, GP40, PA2.26, T1A-2
platelet and endothelial cell adhesion molecule 1	PECAM1	CD31
Urokinase-type plasminogen activator	PLAU	UPA, URK
plasminogen activator, urokinase receptor	PLAUR	CD87, UPAR, URKR
plexin B1	PLXNB1	KIAA0407, PLXN5, SEP
Prominin-1	PROM1	AC133, CD133, CORD12, MCDR2, PROML1, RP41, STG
Receptor-type tyrosine-protein phosphatase eta	PTPRJ	CD148, DEP1, HPTPeta
Resistin	RETN	ADSF, FIZZ3, RETN1
E-selectin	SELE	CD62E, ELAM, ELAM1, ESEL
L-selectin	SELL	CD62L, hLHRc, LAM-1, LAM1, Leu-8, LNHR, LSEL, Lyam-1, LYAM1, PLNHR
sialic acid binding Ig like lectin 1	SIGLEC1	CD169, dJ1009E24.1, FLJ00051, FLJ00055, FLJ00073, FLJ32150, sialoadhesin, SIGLEC-1, SN
sialic acid binding Ig like lectin 10	SIGLEC10	MGC126774, PRO940, SIGLEC-10, SLG2
sialic acid binding Ig like lectin 11	SIGLEC11	
sialic acid binding Ig like lectin 14	SIGLEC14	
Siglec-5	SIGLEC5	CD170, CD33L2, OB-BP2, SIGLEC-5
sialic acid binding Ig like lectin 6	SIGLEC6	CD327, CD33L, CD33L1, OB-BP1, SIGLEC-6
sialic acid binding Ig like lectin 7	SIGLEC7	CD328, p75/AIRM1, QA79, SIGLEC-7, SIGLEC19P, SIGLECP2
Siglec-8	SIGLEC8	MGC59785, SAF2, SIGLEC-8, SIGLEC8L
sialic acid binding Ig like lectin 9	SIGLEC9	CD329
Tumor-associated calcium signal transducer 2	TACSTD2	EGP-1, GA733-1, M1S1, TROP2
Teneurin-1	TENM1	ODZ1, ODZ3, TEN-M1, TEN1, TNM
Teneurin-2	TENM2	KIAA1127, ODZ2, Ten-M2, TEN2
Teneurin-4	TENM4	KIAA1302, ODZ4, Ten-M4, TEN4
CD90/Thy-1 membrane glycoprotein	THY1	CD90
toll like receptor adaptor molecule 2	TICAM2	TICAM-2, TIRP, TRAM
Hepatitis A virus cellular receptor 2	TIM3	CD366, FLJ14428, Tim-3, TIM3, TIMD3
TIMP metallopeptidase inhibitor 2	TIMP2	CSC-21K
Transmembrane protein 204	TMEM204	C16orf30, CLP24, FLJ20898
Trophoblast glycoprotein	TPBG	5T4, 5T4-AG
UL16 binding protein 1	ULBP1	RAET1I
UL16 binding protein 2	ULBP2	RAET1H
UL16-binding protein 3	ULBP3	RAET1N
Vascular cell adhesion protein 1	VCAM1	CD106

S15

No.	Group name	Sex	Age
1	Healthy Control	Female	57
2	Healthy Control	Female	40
3	Healthy Control	Male	71
4	Healthy Control	Female	54
5	Healthy Control	Female	36
6	Healthy Control	Female	52
7	Healthy Control	Female	54
8	Healthy Control	Male	54
9	Healthy Control	Female	32
10	Healthy Control	Female	55
11	Healthy Control	Female	56
12	Healthy Control	Female	35
13	Healthy Control	Male	40
14	Healthy Control	Male	26
15	Healthy Control	Female	38
16	Healthy Control	Male	67
17	Healthy Control	Female	56
18	Healthy Control	Female	38
19	Healthy Control	Female	65
20	Healthy Control	Female	60
21	precancerous lesions	Male	80
22	precancerous lesions	Male	83
23	precancerous lesions	Female	65
24	precancerous lesions	Male	73
25	precancerous lesions	Male	58
26	precancerous lesions	Female	60
27	precancerous lesions	Male	64
28	precancerous lesions	Male	43
29	precancerous lesions	Male	50
30	precancerous lesions	Female	66
31	precancerous lesions	Male	66
32	precancerous lesions	Female	81
33	precancerous lesions	Male	73
34	precancerous lesions	Female	66
35	Stage I CRC	Male	66
36	Stage I CRC	Female	76
37	Stage I CRC	Male	61
38	Stage I CRC	Male	50
39	Stage I CRC	Male	67
40	Stage I CRC	Male	69
41	Stage I CRC	Male	57
42	Stage I CRC	Male	65
43	Stage I CRC	Female	66
44	Stage I CRC	Male	63
45	Stage I CRC	Male	69
46	Stage II CRC	Male	50
47	Stage II CRC	Male	49
48	Stage II CRC	Female	59
49	Stage II CRC	Female	51
50	Stage II CRC	Male	78
51	Stage II CRC	Male	62
52	Stage IV CRC	Male	57
53	Stage IV CRC	Male	62
54	Stage IV CRC	Male	67
55	Stage IV CRC	Male	63
56	Stage IV CRC	Female	56
